# A multi-layer similarity approach for analyzing ADHD symptomology and assessment methods considering DSM-5 diagnostic criteria

**DOI:** 10.3389/fpsyt.2025.1671747

**Published:** 2026-01-26

**Authors:** Syeda Aneela Zahra Shamsi, Zamir Hussain, Mehwish Zaman

**Affiliations:** 1 School of Interdisciplinary Engineering and Sciences (SINES), National University of Sciences & Technology (NUST), Islamabad, Pakistan; 2 Department of Statistical Science, University of Padua, Padova, Italy

**Keywords:** ADHD, ADHD rating scale, DSM-5, similarity layers, SNAP-IV

## Abstract

**Aim:**

Attention-Deficit-Hyperactivity-Disorder (ADHD) is a neurodevelopmental-condition characterized by two symptom-domains, inattention and hyperactivity/impulsivity, as per DSM-5. Prior research, indicates conceptual-overlap among symptoms within each domain, potentially compromising the diagnostic utility of symptom structure itself. This structural redundancy has direct implications for evaluation of ADHD-screening-tools, which already show substantial heterogeneity in item-content and focus. While full psychometric-validation is resource-intensive, assessing tool alignment with DSM-5 offers a more practical and clinically relevant alternative.

**Method:**

Considering these challenges, this study first employed a three-layer-similarity-framework with entropy-based-weighted-combined-score, to investigate intra-domain symptom redundancy. Subsequently, a multi-stage-classification-pipeline, comprising a filtering-layer and machine-learning-classifiers (Random-Forest, Support-Vector-Machine and Logistic-Regression), was trained on DSM-5 ADHD and Non-ADHD (Conduct-Disorder, Major-Depressive-Disorder, Oppositional-Defiant-Disorder) statements, tested on Vanderbilt-preschool-assessment-questionnaire and validated on ADHD-Rating-Scale, Swanson-Nolan-and-Pelham-Rating-Scale (SNAP-IV) and Modified-Checklist-for-Autism-in-Toddlers (M-CHAT), to assess screening-tool’s alignment with DSM-5.

**Results:**

The results revealed moderate-overlap between symptom-pairs (2 and 5) and (5 and 7) within the inattention-domain, with similarity-scores of 0.62 and 0.58 respectively. The filtering-layer demonstrated high accuracy of 97%, perfect precision and specificity in isolating ADHD symptoms. Among classifiers, Random-Forest achieved the best performance with 92% accuracy, 83% precision, 100% recall and 91% F1-score. Validation with ADHD-Rating-Scale ensured near-perfect classification due to its focused symptom set, while SNAP-IV’s inclusion of non-ADHD-items slightly reduced subtype specificity. M-CHAT validation further confirmed the designed pipeline’s ability to exclude non-ADHD symptoms, supporting its classification precision.

**Conclusion:**

The proposed pipeline can be adopted for analyzing strength and limitations of screening-tools, which serve as a catalyst for refinements, ensuring reliability and effectiveness in practical applications.

## 1 Introduction

Attention Deficit Hyperactivity Disorder (ADHD) is a neurodevelopmental condition characterized by a consistent pattern of inattention and hyperactivity-impulsivity that significantly impairs academic, occupational and social functioning (1). According to a meta-analysis conducted in 2023, the worldwide prevalence of ADHD in children and adolescents is estimated to be around 8% (2). As there are no definitive biological markers or objective tests currently available for ADHD, behavioral assessment remains the primary method of diagnosis. In this context, the Diagnostic and Statistical Manual of Mental Disorders, Fifth Edition (DSM-5) provides standardized diagnostic criteria that guide clinical evaluation. According to DSM-5 ADHD is categorized into two core domains: inattention and hyperactivity-impulsivity, each consisting of nine distinct behavioral symptoms (3). Despite this standardized framework, concerns persist regarding the conceptual distinctiveness and item-level redundancy of these symptoms. For instance, symptoms such as “often fidgets with hands or feet”, “is often on the go” and “often leaves seat in situations when remaining seated is expected” may all reflect variations of psychomotor hyperactivity. Likewise, inattention symptoms such as “often loses things necessary for tasks” and “has difficulty organizing tasks and activities” could indicate shared deficits in executive functioning (4). Using techniques such as item response theory, machine learning, network analysis and internal consistency evaluation, researchers have consistently identified symptom-level redundancy and differential contribution in prediction of functional impairment. For instances, a study conducted in 2021, employed network analysis and random forest regression on a nationally representative adults sample and identified only a small subset of symptoms, three inattention (“difficulty organizing task and activity”, “does not follow through instructions” and “make careless mistakes”) and one hyperactive (“difficulty engaging in leisure activities”), as central bridge symptom linked to global and domain specific impairment (5). Similarly in 2021, longitudinal network modeling in children and adolescents, found that only a few symptoms such as “is easily distracted”, “has difficulty sustaining attention”, “difficulties following instructions” and “interrupt/intrudes” were consistently central across both parent and teacher reports, and these central symptoms predicted future emotional and behavioral difficulties as effectively as the full 18-symptoms set (6). Item response theory also demonstrated that “easily distracted” provided substantially more diagnostic information than others (7). Subsequent investigations further reinforced this variability by showing that the predictive utility of individual ADHD symptoms differs across developmental stages and impairment domains, with inattention being more predictive of academic impairment and hyperactivity/impulsivity more relevant for social functioning in early childhood (8). Similarly, ROC-based classification was employed in a distinct study, to develop optimized diagnostic algorithms and reported that models limited to impairment-predictive symptoms significantly outperformed DSM-IV criteria in diagnostic efficiency and inter-rater reliability (9). In 2019, finding emerged showing that among over 116,000 valid symptom combinations, only a few symptoms, such as “motoric activity”, “losing thing” and “does not follow instructions” had high centrality and disproportionate influence on diagnostic outcomes (10). Collectively, these findings indicate that the 18-symptoms list, particularly within the hyperactivity-impulsivity domain, may be reducible without substantial loss of diagnostic information, as individual symptoms may encapsulate content that is redundantly represented across multiple current symptoms (4). On the basis of this notion, we hypothesized that, are there some of the 18 symptoms of ADHD that may exhibit misleading language patterns in DSM-5 description, potentially indicating underlying semantic redundancy? To evaluate this, we implemented a three-layer similarity framework aimed at systematically assessing any possible language ambiguity among the symptom descriptions. Similar techniques have also been implemented in other domains such as text summarization, textual similarities and semantic analysis (11–15). However their application to modeling language ambiguity within ADHD symptomology remains novel.

In addition to evaluating the conceptual distinctiveness of DSM-5 ADHD symptoms set, it is also essential to consider how these symptoms are operationalized in real world settings. This is particularly critical given that ADHD increasingly recognized as lifelong neurodevelopmental condition (16). Early identification can pave the way for effective behavior modification, yielding lasting benefits (17). Maximizing these outcomes hinges on screening children at the earliest possible age. Therefore the American Academy of Pediatrics and the Centers for Disease Control and Prevention’s National Center of Birth Defects and Developmental Disabilities recommend in cooperating routine screenings as a part of developmental surveillance to help pediatricians in early identification (18). Over the years, numerous screening tools has been developed for ADHD assessment with respect to different age groups. However our particular focus in on tools designed for use in pediatric population. One of the earliest, the Conner’s Teacher Rating Scale, a 39 item symptom and behavior checklist, was introduced in 1969, followed by the Conner’s Parent Rating Scale consisting of 73 items, in 1970, with revisions in subsequent years to enhance psychometric precision and clinical relevance (19, 20). The SNAP-IV (Swanson, Nolan and Pelhum Rating Scale), is 90 item self-reporting too, introduced in 1985, provide a structured assessment of core attention and behavior regulation difficulties, suitable for both clinical use and research (21). Later, in 2003, the Vanderbilt ADHD diagnostic Rating Scale (VADRS) was developed to offer a comprehensive screening tool incorporating symptom ratings as well as items related to academic performance and behavioral concerns (22). These tools have become foundational in both educational and clinical practice for identifying children at risk. Additionally, the ADHD Rating Scale has been extensively used due to its alignment with clinical observations and its application in both research and clinical settings for symptoms quantification (23). Beyond these, several other tools have emerged, including the Brown Attention Deficit Disorder Scales, Child Behavior Checklist and Behavior Assessment System for Children, etc. each varying in scope and purpose (24–26). The evolution of ADHD screening tools has been instrumental in early screening, yet the heterogeneity in the assessment pool presents a significant challenge in determining which tool is most effective. Broadly, two approaches exist for evaluating these tools, either assessing their psychometric properties (such as reliability, validity, and sensitivity) or determining their alignment with established diagnostic criteria i-e DSM-5. While evaluating psychometric properties requires extensive validation across diverse populations, assessing tools based on criteria fulfillment provides a more direct and structured method. To maintain clinical relevance, ADHD screening tools must integrate the diagnostic framework, ensuring a more comprehensive assessment. This includes mapping existing questionnaires, validating them against contemporary diagnostic standards. Mapping items to DSM-5 diagnostic criteria is crucial for ensuring the reliability and validity of screening tool. This technique ensure that each item precisely measure the intended construct, leading to more consistence outcomes. Despite the critical role of standardized diagnostic criteria for ADHD assessment, to the best of our knowledge, no similar studies have been conducted to rigorously map screening tools to established frameworks such as DSM-5. This represents a significant gap in the field. Therefore, a multi-level symptom representation and classification pipeline employed to systematically align questionnaire items with DSM-5, enabling a precise evaluation of the strengths and limitations of ADHD screening tools. Similar approach has been used for question, text and fake new classification separately (27–29). The major contribution of this study includes:

- A three layer similarity framework has been designed to examine the coherence and distinctiveness of diagnostic items beyond statistical associations.
- A multi-level symptom representation and classification pipeline has been developed using linguistic features to differentiate ADHD relevant versus non-ADHD symptomatology, followed by subtype classification (inattention vs. hyperactivity-impulsivity).
- Developed pipeline has been validated using three widely adopted tools (e.g., Vanderbilt, SNAP-IV and ADHD Rating Scale). This process identified strengths, inconsistencies and potential misclassifications, particularly where tools blur boundaries between ADHD and comorbid conditions like conduct disorder.

## 2 Methodology

### 2.1 Corpus

To investigate linguistic overlap among the ADHD symptom criteria, this study utilized the set of 18 core diagnostic statements outlined in DSM-5, comprising nine inattention and nine hyperactivity/impulsivity symptoms. Only the primary symptom descriptors were retained, excluding the illustrative examples that accompany each symptom in the manual. This approach allowed for a focused assessment of the conceptual structure and potential semantic overlap within the core diagnostic criteria, as summarized in **Table 1**.

**Table 1:** Core ADHD symptom statements used in the analysis.

Sr. No	Inattention	Hyperactivity/Impulsivity
1	Often fails to give close attention to details or makes careless mistakes	Often fidgets with or taps hands or feet or squirms in seat
2	Often has difficulty in sustaining attention in tasks or play activities	Often leaves seat in situations when remaining seated is excepted
3	Often does not seems to listen when spoken to directly	Often runs about or climb in inappropriate situations
4	Often does not follow through on instructions and fails to finish tasks	Often unable to play or engage in leisure activities quietly
5	Often has difficulty organizing tasks and activities	Is often “on the go” acting as if “driven by a motor”
6	Often avoid of is reluctant to engage in tasks that required sustain mental effort	Often talks excessively
7	Often loses things necessary for tasks and activities	Often blurts out answers before a question has been completed
8	Is often easily distracted by extraneous stimuli	Often has difficulty waiting their turn
9	Is often forgetful in daily activities	Often interrupts or intrudes on other

This table listed the 18 primary ADHD diagnostic symptom statements sourced directly from the DSM-5. Symptoms are categorized into two domains: Inattention and Hyperactivity/Impulsivity. Only the core symptom descriptions were included in the analysis, excluding supplementary examples.

### 2.2 Semantic similarity assessment of ADHD symptom statement

To investigate potential linguistic overlap within ADHD symptom domains, a semantic similarity analysis was conducted. This approach evaluates whether symptom statements convey closely related meanings, despite being treated as distinct diagnostic indicators. For this task, a pre-trained sentence transformer model, pritamdeka/S-Biomed-Roberta-snli-multinli-stsb, was utilized as primary model due to its domain-specific training on biomedical and clinical text, making it particularly suitable for capturing semantic nuances in health-related text (30). While alternative models such as BioBERT or ClinicalBERT are highly effective for token level tasks (e.g. Named Entity Recognition), they often underperform in semantic similarity tasks because they typically rely on simple mean pooling of token embedding, which can dilute overall sentence meaning (31, 32). In contrast the selected primary sentence transformer model employs a Siamese Network Architecture and specifically fine-tuned on Natural Language Inference (NLI) and Semantic Textual Similarity (STS) datasets, making it optimized for computing pairwise cosine similarity of diagnostic statements.

**Figure 1:**
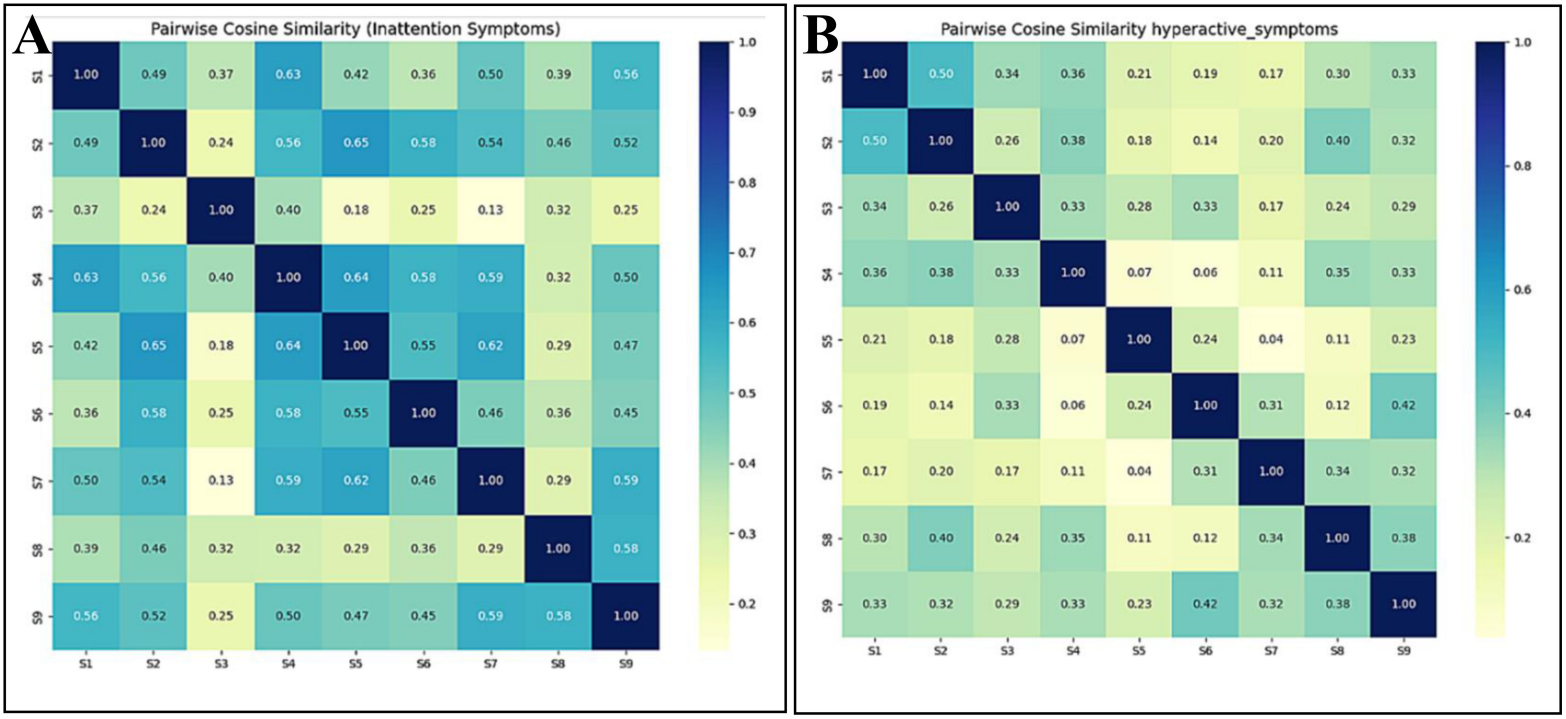
Semantic similarity heat maps for ADHD symptom domains based on sentences embedding. **(A)** Inattention domain: pairwise cosine similarity among symptom statements. **(B)** Hyperactivity/Impulsivity: pairwise cosine similarity among symptom statements. Darker shades indicate higher conceptual similarity between symptoms.

To evaluate the sensitivity of the proposed semantic similarity analysis to model selection, a comparative analysis between the primary model and a widely used general purpose sentence transformer, all-mpnet-base-v2 (33). Each symptom statement was passed through each model to generate 768-dimentional sentence embedding, that effectively captured their semantic representations. Subsequently, pairwise cosine similarity scores were computed among the embedding within each domain to quantify conceptual proximity between statements. This process has been demonstrated in **Supplementary Figure S1** (Appendix 2). The resulting similarity patterns are illustrated using heat maps in **Figure 1A** (Inattention) and **Figure 1B** (Hyperactivity/Impulsivity) for primary model. While the similarity patterns generated by general purpose model has been given in **Supplementary Figure S2** (Appendix 2). To access the agreement between models, Spearman rank correlation was calculated between the similarity scores generated by general purpose and primary model for each symptom domain. Rank base correlation was chosen to focus on relative ordering rather than absolute similarity values, which are known to vary across embedding spaces.

**Figure 2:**
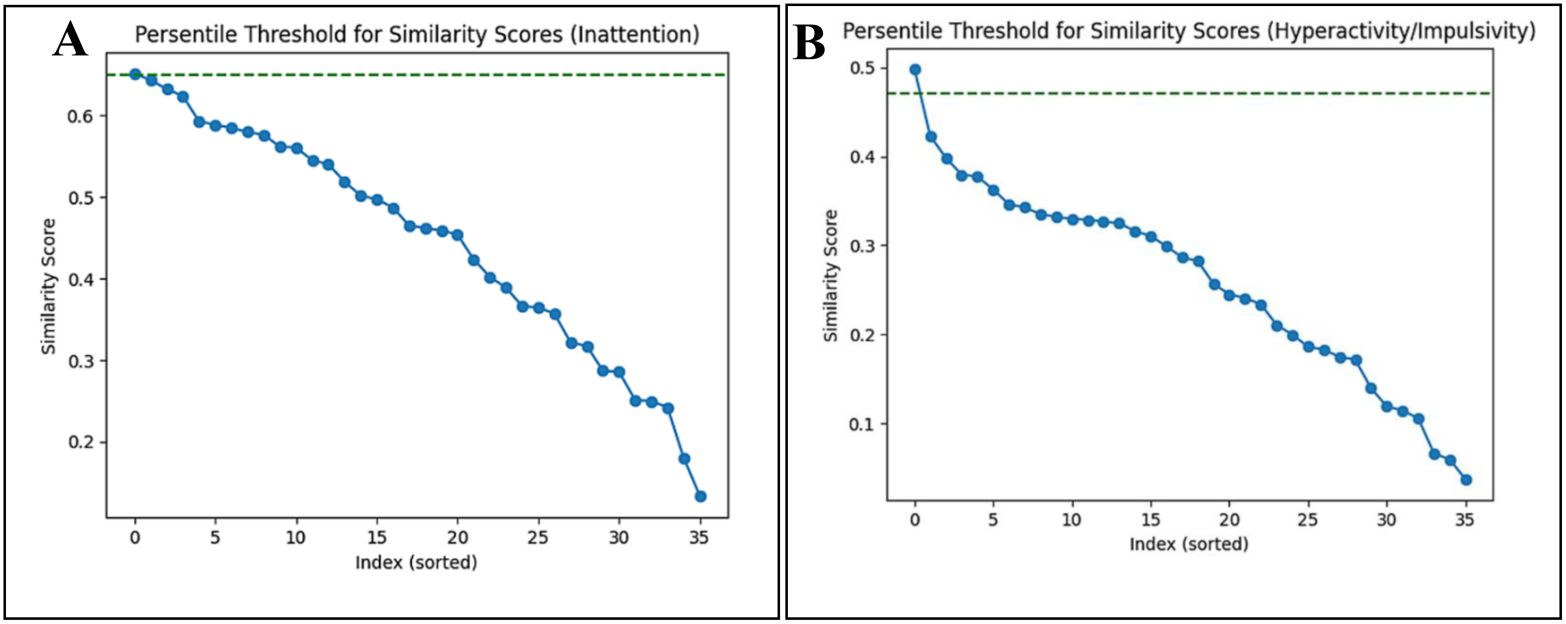
Distribution of pairwise semantic similarity scores within ADHD symptom domains. **(A)** Inattention symptoms and **(B)** Hyperactivity/Impulsivity symptoms. The green dashed lines indicate the top 1^st^ percentile thresholds (0.65 for inattention and 0.47 for hyperactivity/impulsivity), used to identify highly similar symptom pairs.

To systematically identify symptom pairs exhibiting significant linguistic overlap, Rather than adopting an arbitrary universal threshold (e.g., 0.75), a percentile-based approach was implemented to ensure sensitivity to the distributional characteristics of cosine similarity scores within each domain. A threshold of 0.65 (± 3) and 0.79 (± 3) was set for the inattention domain and 0.47 (± 3) and 0.67 (± 3) for hyperactivity/impulsivity domain using primary and general purpose model respectively. These values correspond to approximately the top 1^st^ percentile of the respective similarity distributions, ensuring that only the most semantically proximal pairs were retained for further interpretation. The relaxation of ±3 around the cutoff allows for capturing a small range of values near the threshold to account for natural variability and measurement noise, ensuring that borderline cases with meaningful similarity are not excluded. This percentile-based criterion provides a principled, data-driven approach to distinguish clinically meaningful similarity from general semantic relatedness. This strategy is inspired by prior studies in semantic similarity, text clustering and image thresholding as well (34, 35). The distribution of similarity scores and selected thresholds for both domains using primary model are illustrated in **Figure 2A** (inattention) and **Figure 2B** (Hyperactivity/Impulsivity). While the distribution of similarity scores and selected thresholds using general purpose model is demonstrated in **Supplementary Figure S3** (Appendix 2).

Across symptom domains, similarity scores differed in magnitude and ranking across models, indicating model dependent representation of DSM-5 symptom language. In inattention domain, similarity scores generated by primary and general purpose model showed moderate rank agreement (Spearman ρ 0.54 (p-value: 0.000)) and percentile-based-selection revealed partial overlap, with the general purpose model identifying three highly similar symptom pairs, all of which were also selected by the primary model, which identified one additional pair. In contrast, for hyperactivity/impulsivity domain, rank agreement was slightly higher (Spearman ρ 0.63 (p-value: 0.000)), yet threshold based selection slightly diverged, with the general purpose model identifying three pairs while primary model identifying a single pair, with no overlap between the selected set. Qualitative inspection at granularity level (symptom pairs) further indicate that the general purpose model occasionally assigned relatively high scores to clinically related but conceptually distinct behaviors (e.g. pair (3,6) in hyperactivity/impulsivity domain), where the primary model produced more conservative similarity estimates for such pairs as shown in **Table 2** and **Supplementary Table S1** (Appendix 2). Although no external clinical ground truth was used to established absolute correctness, these differences are consistent with the respective training objectives of the model and highlight that semantic similarity analysis of DSM-5 symptom descriptions are inherently model dependent. On this basis, the pritamdeka/S-Biomed-Roberta-snli-multinli-stsb model was retained as primary model for subsequent analysis due to its closer alignment with clinically oriented sentence level distinction.

**Table 2:** Symptoms pairs exhibiting high sematic similarity based on cosine scores within each ADHD domain.

Sr.no	Pair	Symptom 1	Symptom 2	Similarity score	Domain
1	(1,4)	Often fails to give close attention to details or makes careless mistakes	Often does not follow through on instructions and fails to finish tasks	0.63	Inattention
2	(2,5)	Often has difficulty in sustaining attention in tasks or play activities	Often has difficulty organizing tasks and activities	0.65	Inattention
3	(4,5)	Often does not follow through on instructions and fails to finish tasks	Often has difficulty organizing tasks and activities	0.64	Inattention
4	(5,7)	Often has difficulty organizing tasks and activities	Often loses things necessary for tasks and activities	0.62	Inattention
5	(1,2)	Often fidgets with or taps hands or feet or squirms in seat	Often leaves seat in situations when remaining seated is excepted	0.50	Hyperactivity/Impulsivity

Four pairs were identified in inattention and one in hyperactivity/impulsivity domain respectively.

To conceptualize the percentile-based threshold strategy, the primary sentence transformer model was additionally evaluated on the Semantic Textual Similarity Benchmark (STS-B), a standard dataset containing human-annotated sentence similarity scores normalized between 0 and 1 (36). On STS-B, cosine similarity scores produced by the model showed very strong alignment with human-judgments, yielding a Spearman correlation of approximately 0.96 between model’s generated similarities and gold standard annotations as shown in **Supplementary Figure S4** (Appendix 2). At higher similarity ranges, sentence pair within the top 95-99^th^ percentile exhibited cosine similarity value above 0.96, with corresponding human similarity scores exceeding 0.95, indicating near phrase-level agreement. Importantly, because similarity values observed in the ADHD symptom analysis predominately lay within a moderate range (0.50 to 0.65) (shown in **Table 2**), model behavior was specifically examined at comparable similarity levels in STS-B. At mid-range similarity values (cosine similarity = 0.50 to 0.65), the mean human annotated similarity score was 0.55 with a median of 0.56, closely matching the model generated values at this range and indicating reasonable calibration beyond only extreme similarity cases. While STS-B does not provide clinical validation for DSM-5 symptom comparisons, this external benchmarking supports the use of percentile-based data-driven thresholds as a principled method for identifying relative degree of semantic similarity within a constrained symptom set.

Despite moderate semantic similarity scores, a deeper functional examination reveals important diagnostic distinctions. For instance, the inattention symptoms *“often fails to give close attention to details or make careless mistakes”* and *“often does not follow through on instructions and fails to finish tasks”* scored 0.63 on semantic similarity. However, the first symptom pertains primarily to selective attention and momentary lapses in cognitive processing, whereas the second involves deficits in working memory that linked with executive functioning (37, 38). Similarly, in hyperactivity/impulsivity domain, the symptoms *“often fidgets with or tap hands or feet or squirms in seat”* and *“often leaves seat in situations when remaining seated is expected”* show a semantic similarity of 0.50, yet represent distinct behavioral contexts. One reflecting fine motor restlessness or excessive non-goal motor movement, the other indicating context-inappropriate behavioral inhibition (39, 40). These observations underscore a key limitation of relying solely on semantic similarity, as embedding models can conflate distinct constructs when symptoms share similar contextual language. This overlap is especially problematic in clinical settings, where superficially alike wording may reflect fundamentally different functional impairments. Therefore, to improve the resolution of similarity analysis and minimize false conceptual overlap, lexical and syntactic features has been incorporated in further analysis. Lexical features identify subtle vocabulary differences, while syntactic patterns reflect variations in behavioral structure.

### 2.3 Lexical similarity assessment of ADHD symptom statement

#### 2.3.1 Preprocessing

To prepare the symptom statements for lexical similarity analysis, a preprocessing pipeline was implemented to standardize and clean the text related to both domains (inattention and hyperactivity/impulsivity) separately. Each sentence was first converted to lowercase to ensure case insensitivity, followed by tokenization using NLTK’s “word-tokenize” method, which segments the sentence into individual word tokens. Common English stop words, such as articles, conjunctions and auxiliary verbs, were removed to eliminate function words that do not contribute meaningful semantic content. Additionally, all tokens were filtered to retain only alphabetical characters, excluding punctuation and special symbols. The remaining tokens were lemmatized using the “Word Net Lemmatizer”, which reduces each word to its base or dictionary form (e.g., “running” becomes “run”), helping to group morphological variants under a single representative form. Before lemmatization, Part-Of-Speech (POS) tagging was applied to each token to identify its grammatical role within the sentence. This step is essential for enabling context-aware lemmatization, as the lemmatizer requires explicit POS input to accurately reduce words to their base forms. In the absence of POS tagging, standard lemmatizer such as “Word Net” default to noun-based transformations, which can result in incorrect lemmatization. For instance, in the symptom *“often leaves seat when remaining seated is expected”*, the word “leaves” is a verb, however, without POS guidance, lemmatizer treat it as noun and it would incorrectly be reduced to “leaf”. By assigning the correct POS tag, verb in this case, lemmatizer accurately transforms “leaves” into its root form “leave”. Each token was thus first POS tagged using the Penn Treebank tag set and then mapped to Word Net compatible POS categories (i.e., noun, verb, adjective and adverb) prior to lemmatization. This preprocessing ensures that the lexical similarity measures are based on meaningful content words, enhancing the accuracy and interpretability of subsequent similarity computations.

#### 2.3.2 Lexical similarity calculation

Following preprocessing, lexical similarity between pairs of symptom statements were computed using a word-to-word alignment framework adopted from prior work in lexical sematic analysis (11). For each token pair between two sentences, similarity was calculated using a hybrid method. First, path-based semantic similarity was computed using Word Net’s path distance, which reflects the conceptual proximity between two words in the lexical ontology. If no valid semantic path existed between the words, or if the computed similarity was below a minimum threshold (0.1), a fallback levenshtein similarity was used. This string-level metric captures the degree of surface similarity between two words based on character-level edits. Using these measures, a word-level similarity matrix was constructed for each sentence pair, where each cell represents the similarity between a token from the first sentence and a token from the second. The total similarity between two sentences was computed using a greedy alignment algorithm. At each iteration, the highest remaining similarity score in the matrix was selected and added to a cumulative total. The corresponding row and column were then removed from further consideration to avoid reusing aligned tokens. This process was repeated until all row or columns had been exhausted. The final word similarity was obtained by dividing the accumulated score by the number of iterations (i.e., the number of aligned word pairs). To account for asymmetry in sentence length, a penalty term was applied. The penalty was computed as the absolute difference between the token lengths of the two sentences, multiplied by the computed similarity, and divided by the maximum of the two sentence lengths. This penalty was subtracted from the word similarity score to obtain a length-normalized similarity and this score is considered final lexical score. A detailed example illustrating this entire process is provided in Appendix 1.

### 2.4 Syntactic similarity assessment of ADHD symptom statement

#### 2.4.1 Preprocessing

In the syntactic similarity layer, preprocessing is limited to the removal of punctuation marks from all symptom sentences related to both domains (inattention and hyperactivity/impulsivity) separately. This step eliminates structurally irrelevant tokens that commonly appear as “punct” dependencies, which has no meaningful contribution to the grammatical structure of the sentence. The rest of the sentence is retained in full to preserve its syntactic structure, as function words such as auxiliaries and prepositions are essential for accurate dependency parsing. This allows for the accurate extraction of grammatical relations necessary for constructing RDF-style syntactic triples, which form the basis of the syntactic similarity computation. Additionally all tokens are converted to lowercase and lemmatized to ensure consistent word forms and improve the matching syntactic elements across sentences.

#### 2.4.2 Syntactic similarity calculation

**Figure 3:**
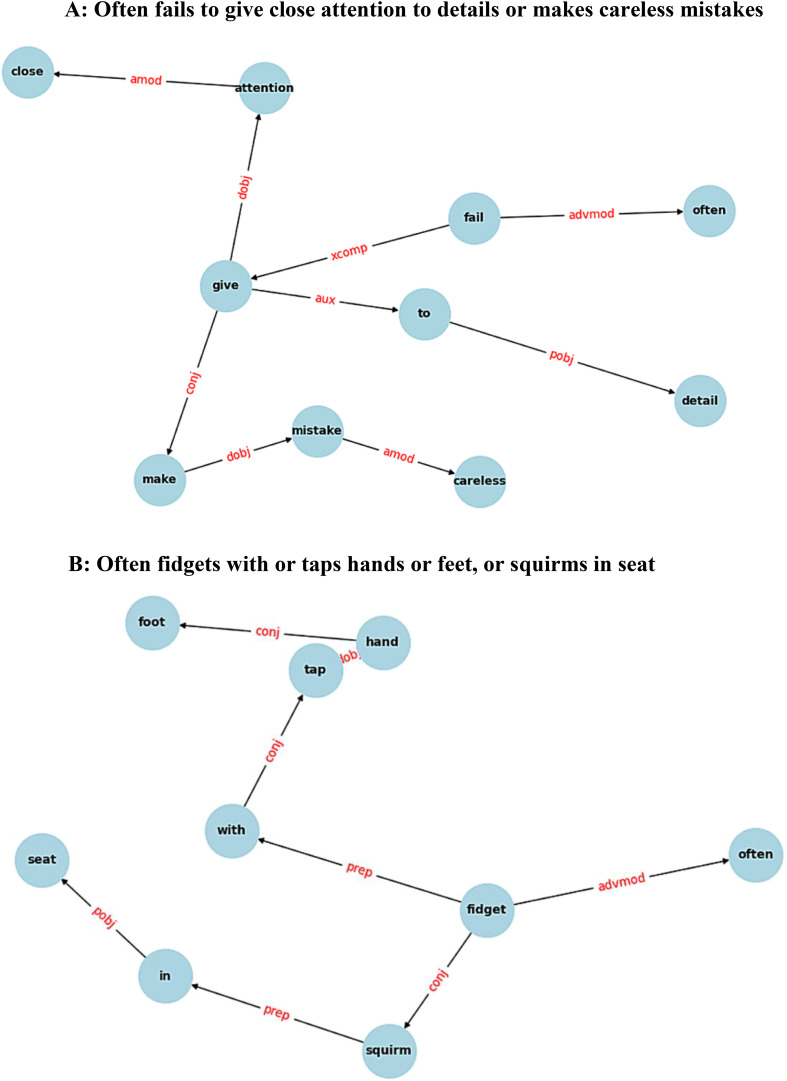
Full syntactic dependency graphs of **(A)** inattention **(B)** hyperactivity/impulsivity symptom 1 respectively. Each graph represents the complete syntactic structure of the sentence, with nodes as lemmatized tokens and edges as grammatical relations, based on filtered dependency types.

The syntactic triple extraction method used in this study follows the approach proposed in (11), which represents sentence structure using RDF-style dependency triples. After preprocessing, each sentence is parsed using spaCy’s dependency parser to extract syntactic relations in the form of head, relation and dependent. These triples encode the grammatical relationships between tokens and capture the core syntactic structure of the sentence. To enhance the relevance of the extracted structure, only a selected subset of dependency types is retained. For instance nominal subjects, direct objects, indirect objects, adverbs, prepositions and auxiliary verbs etc. This filtering removes low impact dependencies such as determinants, which contribute minimally to structural similarity. Dependency graph examples resulting from this process are illustrated in **Figure 3**, and representative triples from inattention symptoms 1 and 4 are shown in **Table 3**. After extracting syntactic triples from each sentence, pairwise similarity between triples was computed using the same hybrid method defined in the lexical layer. However, instead of comparing individual tokens, the comparison has been performed between syntactic triples by evaluating the similarity between their corresponding vertices. For each pair of triples, two alignment scores has been calculated: the first by averaging the word similarity between corresponding head and dependent words, and the second by averaging the similarity in a cross-wise manner (i.e., head to dependent and dependent to head). The final similarity between two triples was obtained by averaging these two alignment scores. This approach allows for both direct and cross alignment between the vertices of the two triples, improving robustness against syntactic variation. Using this formula, a similarity matrix was constructed where each cell represents the similarity score between a triple from the first sentences and one from the second. A greedy alignment algorithm, identical to that used in the lexical layer, was then applied to aggregate these scores into an overall syntactic similarity between sentences. This algorithm selects the best-matching pairs of triples without repetition and averages their scores, yielding the final syntactic similarity value. A detailed example illustrating this entire process is provided in Appendix 3.

**Table 3:** Representative syntactic triples extracted from inattention domain symptom 1 and 4.

Triples of symptom 1	Triples of symptom 4
(‘fail’, ‘advmod’, ‘often’)	(‘follow’, ‘advmod’, ‘often’)
(‘give’, ‘aux’, ‘to’)	(‘follow’, ‘aux’, ‘do’)
(‘fail’, ‘xcomp’, ‘give’)	(‘follow’, ‘neg’, ‘not’)
(‘attention’, ‘amod’, ‘close’)	(‘follow’, ‘prep’, ‘on’)
(‘give’, ‘dobj’, ‘attention’)	(‘on’, ‘pobj’, ‘instruction’)
(‘to’, ‘pobj’, ‘detail’)	(‘follow’, ‘conj’, ‘fail’)
(‘give’, ‘conj’, ‘make’)	(‘finish’, ‘aux’, ‘to’)
(‘mistake’, ‘amod’, ‘careless’)	(‘fail’, ‘xcomp’, ‘finish’)
(‘make’, ‘dobj’, ‘mistake’)	(‘finish’, ‘dobj’, ‘task’)

Each triple is in the form (head, relation, dependent) and reflects a meaningful grammatical relationship identified by the dependency parser.

### 2.5 Validation of multi-layer similarity assumption

After computing similarity scores across semantic, lexical and syntactic layers for all symptom pairs, next step involved evaluating the relationship between these layers. While semantic similarity particularly when derived from Sentence Transformers, is often assumed to be sufficient for capturing overall meaning (41). It remain important to access whether it sufficiently reflects other linguistic dimensions because this may not hold in contexts where fine-grained linguistics cues play a significant role. To investigate this, two complementary statistical methods were employed to assess whether lexical and syntactic similarities provide non-redundant, complementary information beyond what is captured semantically.

#### 2.5.1 Wilcoxon signed-rank test

To determine whether lexical and syntactic similarity scores differ significantly from semantic similarity scores, the Wilcoxon signed-rank test was used (42). This non-parametric test is designed for comparing paired samples and evaluates whether the median of the differences between them is zero. It is suitable in this context as it does not assume normality and is robust to the skewed distribution of similarity values. The test was applied separately for the inattention and hyperactivity/impulsivity symptom domains, results has been illustrated in **Table 4**. Statistically significant results in all cases (p-value< 0.05) confirm that lexical and syntactic similarities differs meaningfully from semantic similarity. This indicates that the semantic layer does not entirely capture the information encoded at the lexical and syntactic levels.

**Table 4:** Results of Wilcoxon signed-rank test and mutual information regression comparing semantic similarity with lexical and syntactic similarity across inattention and hyperactivity/impulsivity domain.

Wilcoxon signed-rank test			
Domain	Comparison layers	Test statistic	p-value
Inattention	Semantic vs. Lexical	141.0	0.002
	Semantic vs. Syntactic	79.0	0.000
Hyperactivity/Impulsivity	Semantic vs. Lexical	88.0	0.000
	Semantic vs. Syntactic	86.0	0.000

Mutual information regression			
Domain	Comparison layers	Mutual information value	
Inattention	Semantic vs. Lexical	0.009	
	Semantic vs. Syntactic	0.000	
Hyperactivity/Impulsivity	Semantic vs. Lexical	0.1746	
	Semantic vs. Syntactic	0.2991	

#### 2.5.2 Mutual information regression

To further examine the dependence between similarity layers, mutual information regression was employed (43). Mutual information quantifies the amount of information share between two variables, capturing both linear and non-linear associations. In this context, it measure how much knowledge of the lexical and syntactic similarity scores reduce uncertainty about semantic similarity scores. A value of zero indicates complete independence, while higher values suggest stronger dependency. The results for mutual information regression is given in **Table 4**. These results provide additional support to the findings from the Wilcoxon test. In particular, the zero mutual information in the hyperactivity/impulsivity domain between syntactic and semantic similarity indicates statistical independence, confirming that synaptic patterns are not captured by semantic representation. Even in the inattention domain, while mutual information values are slightly higher from zero, suggesting partial overlap but not redundancy.

Together these analysis strongly support the claim that lexical and syntactic features contribute unique and necessary information when assessing overlap in clinical symptom descriptions.

### 2.6 Entropy-based feature weighing

In this study, Entropy Weight Method (EWM), a widely recognized technique within the Multiple Criteria Decision Making (MCDM) framework, has been employed to objectively determine the weights of different similarity layers without requiring any prior knowledge or labeled data (44, 45). The method leverages the concept of entropy from information theory to quantify the degree of disorder or uncertainty in the distribution of features values. Features exhibiting greater variability and lower entropy carry more informative content and are thus assigned higher weights. This approach facilitates an unbiased weighting process driven solely by the data itself, making it especially suitable for unsupervised scenarios. The procedure consists of the following steps:

1. Data normalization: Each similarity layer values has been normalized such that the sum of its values across all samples equals one. This was achieved by dividing each value in the feature column (e.g., lexical) by the total sum of that feature. This step converts the original data into a probability distribution for each similarity layer, which is a pre-requisite for computing entropy.

2. Zero value adjustment: To avoid the mathematical undecidedness associated with logarithms of zero during entropy calculation, any zero entries in the normalized data are substituted with a small positive constant, typically $\;10^{-10}$.

3. Computation of normalization factor (k): The normalization factor k ensures that calculated entropy values are scaled between zero and one, making them comparable across different features.


$$k=\frac{1}{\text{ln}\left(m\right)}$$

Where m is the number of samples in dataset and “ln” denoted natural logarithm.

4. Entropy computation: For each similarity layer, entropy has been computed as a measure of uncertainty based on its normalized value distribution by using formula given as:


$$H_{j}=-k\sum_{i=1}^{m}p_{ij}\text{ln}\left(p_{ij}\right)$$

5. Determination of Divergence: The informational contribution of each similarity layer has been represented by its degree of divergence, calculated as one minus the corresponding entropy value. This metric highlights similarity layers with higher variability and information content.

6. Normalization to obtain weights: The divergence values were normalized so that their sum equals one, yielding the final set of similarity layers weights.


$$w_{j}=\frac{1-H_{j}}{\sum_{j=1}^{n}1-H_{j}}$$

These weights reflects the relative importance of each layer based on the inherent data characteristics.

By implementing the entropy weight method, we quantitatively capture the intrinsic significance of each layer with respect to each domain separately, thereby enhancing the robustness and validity of the subsequent data analysis and decision making process. Entropy-based weights for lexical, syntactic and semantic similarities were calculated separately for both domains (inattention and hyperactivity/impulsivity). Lexical similarity weights were 0.35 and 0.21 indicating moderate importance in both domains. Syntactic weights were low, at 0.16 and 0.04, showing limited contribution, especially in the second domain. Semantic weights were highest at 0.49 and 0.75, highlighting its dominant role, particularly in the hyperactivity/impulsivity domain. After that, the combined score for each instance with respect to each domain, was calculated by multiplying each original similarity layer value by its corresponding weight. These weighted feature values were then summed to produce a single overall similarity score per instances. This weighted aggregation reflects the relative importance of each similarity layer as quantified by entropy. The final combine scores are presented in **Tables 5**, **6** for inattention and hyperactivity/impulsivity domain respectively.

**Table 5:** Combined similarity scores calculated by weighting original features (lexical, syntactic and semantic) values with entropy based weights for inattention domain.

Symptom pairs	Lexical similarity	Syntactic similarity	Semantic similarity	Combined similarity
(1, 2)	0.389	0.387	0.48718	0.437091
(1, 3)	0.269	0.369	0.366129	0.332715
(1, 4)	0.43	0.392	0.632559	0.523859
(1, 5)	0.25	0.411	0.423508	0.361027
(1, 6)	0.407	0.323	0.364275	0.372641
(1, 7)	0.278	0.302	0.501854	0.39217
(1, 8)	0.239	0.295	0.388685	0.321664
(1, 9)	0.222	0.354	0.560764	0.409917
(2, 3)	0.282	0.273	0.24187	0.260789
(2, 4)	0.505	0.363	0.561909	0.510588
(2, 5)	0.619	0.541	0.651628	0.622744
(2, 6)	0.457	0.428	0.584822	0.515434
(2, 7)	0.544	0.347	0.540378	0.51104
(2, 8)	0.3	0.283	0.461771	0.377072
(2, 9)	0.381	0.481	0.519009	0.46487
(3, 4)	0.361	0.379	0.402077	0.384101
(3, 5)	0.344	0.29	0.179868	0.254529
(3, 6)	0.25	0.325	0.250725	0.262226
(3, 7)	0.339	0.285	0.133236	0.229002
(3, 8)	0.389	0.274	0.321337	0.33744
(3, 9)	0.362	0.327	0.249352	0.30092
(4, 5)	0.492	0.361	0.643518	0.545976
(4, 6)	0.37	0.357	0.576413	0.469715
(4, 7)	0.583	0.378	0.592837	0.55541
(4, 8)	0.332	0.297	0.317247	0.319187
(4, 9)	0.361	0.433	0.497243	0.439569
(5, 6)	0.324	0.411	0.545229	0.446845
(5, 7)	0.583	0.448	0.623993	0.581849
(5, 8)	0.355	0.245	0.286773	0.303953
(5, 9)	0.49	0.349	0.465048	0.455385
(6, 7)	0.38	0.358	0.459003	0.415472
(6, 8)	0.231	0.305	0.356927	0.304799
(6, 9)	0.241	0.426	0.453879	0.375236
(7, 8)	0.329	0.26	0.285388	0.296578
(7, 9)	0.444	0.369	0.58874	0.503496
(8, 9)	0.362	0.271	0.579831	0.455002

**Table 6:** Combined similarity scores calculated by weighting original features (lexical, syntactic and semantic) values with entropy based weights for hyperactivity/impulsivity domain.

Symptom pairs	Lexical similarity	Syntactic similarity	Semantic similarity	Combined similarity
(1, 2)	0.497	0.378	0.49817	0.492961
(1, 3)	0.302	0.361	0.342444	0.334745
(1, 4)	0.361	0.332	0.362818	0.361164
(1, 5)	0.321	0.339	0.210753	0.239127
(1, 6)	0.202	0.307	0.186174	0.194478
(1, 7)	0.249	0.286	0.171612	0.192536
(1, 8)	0.255	0.364	0.299014	0.292486
(1, 9)	0.257	0.311	0.328696	0.312958
(2, 3)	0.44	0.394	0.256771	0.300792
(2, 4)	0.383	0.377	0.379405	0.380058
(2, 5)	0.345	0.375	0.182809	0.224698
(2, 6)	0.238	0.375	0.139689	0.169988
(2, 7)	0.307	0.33	0.199761	0.227588
(2, 8)	0.405	0.378	0.398189	0.39878
(2, 9)	0.243	0.319	0.316006	0.300849
(3, 4)	0.417	0.433	0.331869	0.353866
(3, 5)	0.545	0.417	0.282405	0.34293
(3, 6)	0.3	0.342	0.329678	0.323975
(3, 7)	0.424	0.314	0.174931	0.232809
(3, 8)	0.433	0.364	0.24489	0.289185
(3, 9)	0.353	0.319	0.286514	0.301772
(4, 5)	0.429	0.406	0.066521	0.156417
(4, 6)	0.219	0.342	0.059587	0.104622
(4, 7)	0.362	0.329	0.114622	0.175258
(4, 8)	0.369	0.417	0.34614	0.353852
(4, 9)	0.27	0.351	0.326678	0.315819
(5, 6)	0.317	0.338	0.240915	0.260851
(5, 7)	0.402	0.32	0.037393	0.125385
(5, 8)	0.529	0.406	0.105811	0.206791
(5, 9)	0.459	0.417	0.233903	0.288583
(6, 7)	0.367	0.363	0.310563	0.324542
(6, 8)	0.375	0.338	0.119194	0.181777
(6, 9)	0.375	0.327	0.422799	0.408836
(7, 8)	0.36	0.307	0.335182	0.339213
(7, 9)	0.376	0.309	0.324902	0.33494
(8, 9)	0.465	0.321	0.377193	0.39325

### 2.7 Feature extraction based on similarity measures

Second aim of this study is to access the effectiveness of screening tools in accordance with DSM-5 diagnostic criteria by developing a robust classification framework. This classification task involves two categories, inattention and hyperactivity/impulsivity, which were assigned binary labels 0 and 1, respectively. Feature extraction was performed by leveraging similarity scores computed between 18 symptoms describe in **Table 1** (46, 47). Specifically, lexical, syntactic and semantic similarities were measured between each target sentence and two predefined symptom domain corresponding to the assigned classes. For each similarity metric, the average similarity to all sentences within each symptom domain is calculated, and the difference between these averages form the basis of the feature values. This process produces a concise three-dimensional feature vector for each sentence, capturing its relative closeness to both classes across multiple linguistic dimensions. By transforming pairwise similarity information into fixed-length feature representations, this approach facilitates effective binary classification.

### 2.8 Model development and evaluation

The analysis was implemented as two-stage machine learning pipeline, where symptom statements flow sequentially from a filtering stage to downstream classification models. In the first stage a semantic filtering layer was applied to distinguish ADHD consistent symptom language from non-ADHD diagnostic language before further processing. This filtering layer was implemented using a logistic regression classifier trained on DSM-5 diagnostic statements, with ADHD symptoms treated as a positive class and DSM-5 symptoms from Conduct Disorder (CD), Oppositional Defiant Disorder (ODD) and Major depressive disorder (MDD) treated as the negative class. These symptoms were not treated as clinically exclusive to ADHD, as symptom co-occurrence across psychiatric disorders is well-established and explicitly acknowledged. Instead they were used as contrastive examples to model differences in diagnostic language and symptom framing at textual level. ADHD screening instruments are specifically designed to assess core inattention and hyperactivity/impulsivity constructs, therefore introducing non-ADHD DSM-5 symptoms formulations enable examination of whether such tools preferably retain canonical ADHD-related language or admit broader behaviors and affective symptom descriptions. This design choice serve to probe the linguistics specificity and limitations or strengths of ADHD screening tools, rather than to impose categorical diagnostic boundaries. Importantly, all non-ADHD DSM-5 statements were confined to the training phase of the filtering layer and were never used in downstream evaluations. All symptom statements (ADHD and Non-ADHD) were encoded using a fixed pre-trained sentence transformer, pritamdeka/S-Biomed-Roberta-snli-multinli-stsb, generating 768 dimensional semantic embedding that serve as input features to the filtering layer (logistic regression). The logistic regression model is trained with default parameters except for max_iter =1000 to ensure convergence. The training data for filter exhibit class imbalance (18 ADHD vs. 32 Non-ADHD statements), which is formally accessed using a chi-square test (chi-square test statistics = 3.920, p-value = 0.048), indicating statistically significant imbalance at alpha 0.05. To address this, Adaptive Synthetic Sampling (ADASYN) a resampling technique was applied prior to training the filter. This technique was selected because it adaptively shift the decision boundary by generating synthetic samples in difficult regions of the minority class rather than sample duplication, thereby improving class separability without inflating redundant samples (48).

Once trained the filtering layer was applied to test dataset, and only those statements classified as ADHD consistent were passed to the second stage of the pipeline. In the second stage three supervised classifiers were trained exclusively on ADHD DSM-5 statements using the predefined extracted lexical, syntactic and semantic similarity features. These classifiers includes 1) a Logistic Regression model with default parameters 2) a Support Vector Classifier using an RBF kernel with probability estimations enable and 3) a Random Forest classifier with 100 tress and a fixed random state of 42 (49–51). No hyper-parameter tuning was performed at this stage, allowing difference in performance to reflect model characteristics rather than optimization choices. Training data at this stage consists only of DSM-5 ADHD statements, while all screening tools were reserved strictly for testing and validation.

For evaluation, all items from the Vanderbilt Preschool Assessment Questionnaire were first processed through the complete pipeline to access baseline performance of the three classifiers under identical conditions (22). Model evaluation was conducted using standard metrics such as accuracy, precision, recall, F1 score and ROC curve to comprehensively assess classification effectiveness. This combined architecture leverages both embedding-based filtering and feature-based classification to robustly distinguish ADHD symptom profiles.

**Figure 4:**
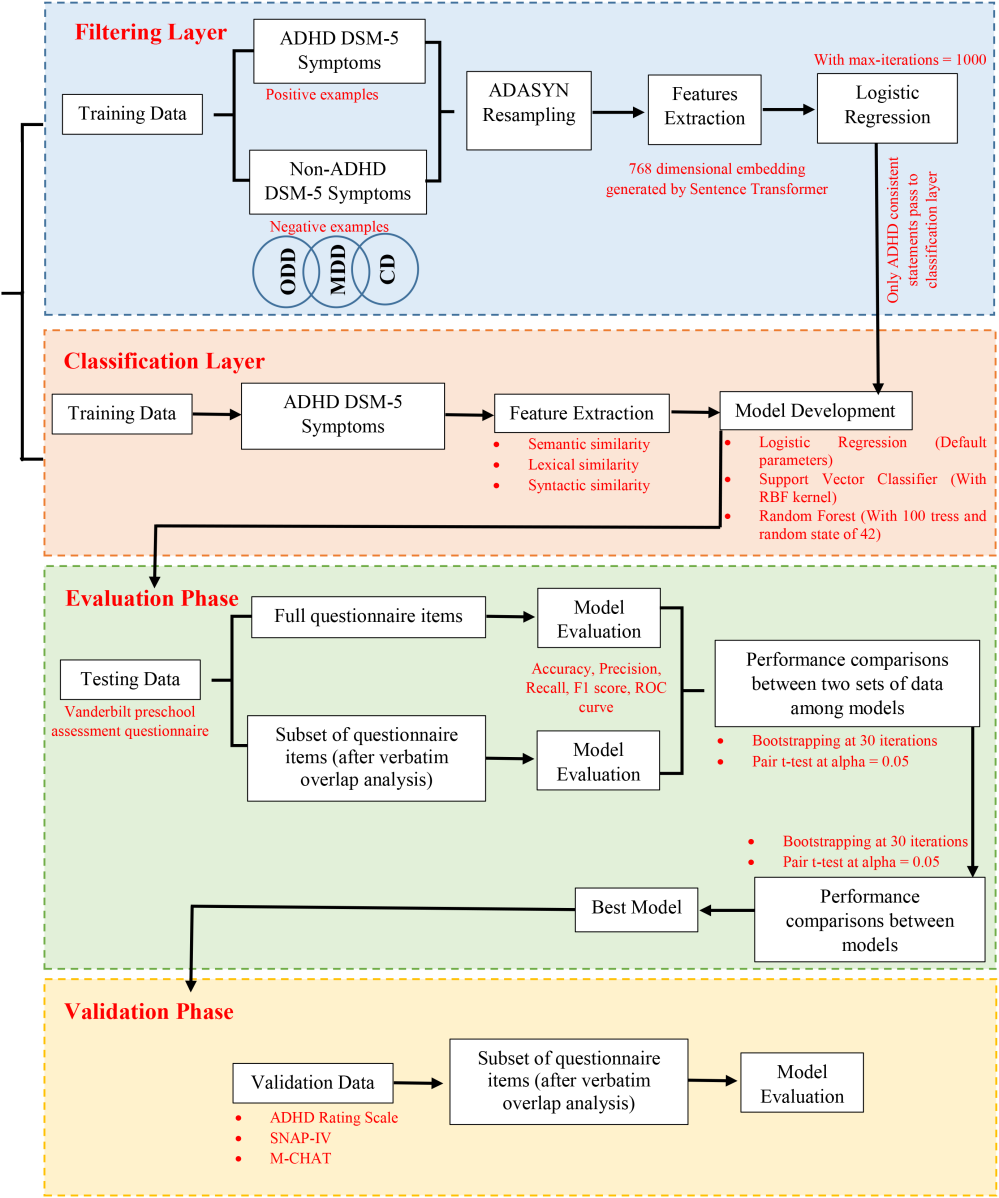
Process flow for model development, evaluation and validation.

Because screening instruments are frequently derived from DSM-5 language, a dedicated data-leakage analysis was then conducted to identify near-verbatim overlap between training and test dataset. Specifically cosine similarity was computed between all DSM-5 sentences used during training (including ADHD and Non-ADHD statements from filtering layer) and all questionnaire items, using a conservative threshold of cosine similarity greater than or equal to 0.90 to identify highly overlapping sentence pairs for near-duplicate or paraphrase equivalent sentences rather than general semantic/topical relatedness (36). This analysis identified approximately 26% of questionnaire items as highly overlapped. These overlapping items were than excluded, and the full pipeline was re-applied to the reduced test set to examine robustness of classifiers performance in the absence of near identical training language. To access this, performance stability was examined using bootstrapping resampling. For both the original (overlap included) and over-lap excluded test sets, bootstrapping resampling with 30 iterations was conducted (choosing owing to small sample size) as shown in **Table 7**. Model accuracy was recalculated at each iteration. Paired t-test were then applied to compare bootstrap accuracy distribution across the two datasets, allowing statistical assessment of whether classifier performance was materially affected by overlapping content. Following the leakage assessment, model selection was performed by comparing three second stage (1^st^ stage is filtering layer and 2^nd^ is classification layer as shown in **Figure 4**) classifiers using the same bootstrap framework. This procedure was applied on overlap excluded test set, and performance distributions were compared between each classifier (e.g. Random Forest vs. Support Vector Machine). Paired t-tests were then used to access statistically meaning full change in performance. This comparison was used to identify the most stable and reliable model for subsequent external validation, while ensuring that observed performance was not driven by textual overlap between diagnostic criteria and screening tool items. Complete flow of pipeline has been shown in **Figure 4**.

**Table 7:** Distribution of samples across the training, test and validation datasets, showing the total number of instance and class proportions in each split.

Datasets	Source	Filter layer	Classification layer
Training	DSM-5 Statements	32 (Non-ADHD)	18 (9=inattention, 9=Hyperactivity/Impulsivity)
Testing (Before Overlap)	Vanderbilt preschool assessment Scale	28 (Non-ADHD)	19 (10=inattention, 9=Hyperactivity/Impulsivity)
Testing (Excluded Overlap)		22 (Non-ADHD)	13 (6=inattention, 7=Hyperactivity/Impulsivity)
Validation (Before Overlap)	ADHD-Rating Scale, SNAP-IV, M-CHAT	31 (Non-ADHD)	36 (18=inattention, 18=Hyperactivity/Impulsivity)
Validation (Excluded Overlap)		26 (Non-ADHD)	25 (13=inattention, 12=Hyperactivity/Impulsivity)

### 2.9 External validation

For external validation, the best performing classifier identified in the previous stage was evaluated on three independent instruments, the ADHD Rating Scale, SNAP-IV and M-CHAT. The ADHD Rating scale and SNAP-IV were selected due to their wide spread clinical use, that differ in item phrases, behavioral emphasis and response structure, that allow examination of how variation in symptom wording influence model behavior. M-CHAT was included as a Non-ADHD screening instrument to access designed pipeline specificity. Prior to evaluation, a verbatim overlap analysis was conducted between the training corpus and validation items using cosine similarity, with a conservative threshold of greater than or equal to 0.90 to identify near duplicate content. Approximately 24% of validation items exceeded this threshold and were excluded. The selected model was then applied to the remaining non-overlapping items, and performance was reported using standard classification metrics to access generalization under strict leakage control. This approach tests the model’s generalizability and specificity across related neurodevelopmental disorders. The use of multiple standardized instruments strengthens the robustness of the validation process.

## 3 Results and discussion

### 3.1 Overlapping symptom pairs based on entropy-weighted combined similarity scores

Using the combined similarity scores, cutoff thresholds were defined at the top 1^st^ percentile ±3 to identify the most similarly related symptom pairs. The 1^st^ percentile cutoff for the inattention domain was set at 0.61 (± 3), while for the hyperactivity/impulsivity domain it was 0.47 (± 3). The relaxation of ±3 around the cutoff allows for capturing a small range of values near the threshold to account for natural variability and measurement noise, ensuring that borderline cases with meaningful similarity are not excluded. Within these thresholds, two pairs of symptoms were identified in the inattention domain and one pair in the hyperactivity/impulsivity domain exhibiting the highest similarity values among their similarity score distribution. The first pair, *“often has difficulty sustaining attention in tasks or play activities”* and *“often has difficulty organizing tasks and activities”*, showed a similarity score of 0.62. The second pair, *“often has difficulty organizing tasks and activities”* and *“often loses things necessary for tasks and activities”*, had a similarity of 0.58 as shown in **Table 5**. In the hyperactivity domain, the pair *“often fidgets with or taps hands and feet, or squirms in seat”* and *“often leaves seat in situations when remaining seated is expected”* demonstrated a similarity of 0.49 as represented in **Table 6**.

Among them, first 2 pairs are more debatable because their similarity scores indicate meaningful but moderate overlap, while the 3^rd^ pair has a low similarity which reflects more clearly distinct symptom presentation. The two symptoms, “often has difficulty sustaining attention in tasks or play activities” and “often has difficulty organizing tasks and activities”, exhibit notable similarity despite representing distinct neurocognitive impairments because of their functional interdependence in goal-directed behavior. Sustained attention is primarily associated with the ability to maintain focused cognitive engagement over time, a function critically mediated by prefrontal cortex circuits regulating vigilance and behavioral control (52). This deficit impairs the capacity to sustain mental effort necessary for task completion. On the other hand, difficulty organizing tasks reflects dysfunction in working memory, planning and executive control process, higher order functions depend on overlapping but partially separable prefrontal and front striatal networks responsible for managing, sequencing and prioritizing information (53, 54). Although impaired working memory and poor planning contribute to disorganization, these processes involve complex manipulation of information beyond pure attentional focus (52, 54). The moderate similarity arises because ineffective sustained attention can exacerbate problems with organization by limiting the individual’s ability to maintain task-relevant information online, creating a downstream impact on planning and task management (53, 55). Empirical data support that while these cognitive domains interact and often co-occur in ADHD, they represent dissociable constructs with distinct neurobiological subtracts (53). Therefore, despite their semantic overlap and functional association, these symptoms map onto different executive domains, vigilance vs. working memory/executive control, and should be interpreted as related but neurocognitive distinct deficits. This distinction has important implications for targeted assessment and intervention in ADHD. While the symptom pair, *“often has difficulty organizing tasks and activities”* and *“often loses things necessary for tasks and activities”*, reflect closely related manifestations of executive dysfunction, particularly impairments in planning, organization, and working memory. Losing things necessary for tasks, such as tools, materials or belongings is commonly viewed as a behavioral consequence of disorganization and working memory deficits, where failure to monitor, update or recall task relevant information impairs task execution. According to Neuropsychological study, these symptoms engage similar prefrontal and front striatal circuits responsible for maintaining and manipulating information during goal directed behavior (56). However, difficulty organizing tasks captures a higher level strategic aspect of executive control, while losing things reflects lapses in real time monitoring and item tracking, implicating aspects of prospective memory and attentional control. Therefore, although both symptoms are strongly interrelated and often co-occur, they represent distinct but complementary facets of executive function deficits commonly observed in ADHD and related conditions (57). Clinically, this distinction is important, interventions targeting organizational skills may differ from those addressing memory strategies to reduce item loss, suggesting these symptoms should be considered closely linked but not identical (58, 59).

### 3.2 Evaluation of Multi-stage classification pipeline

**Figure 5:**
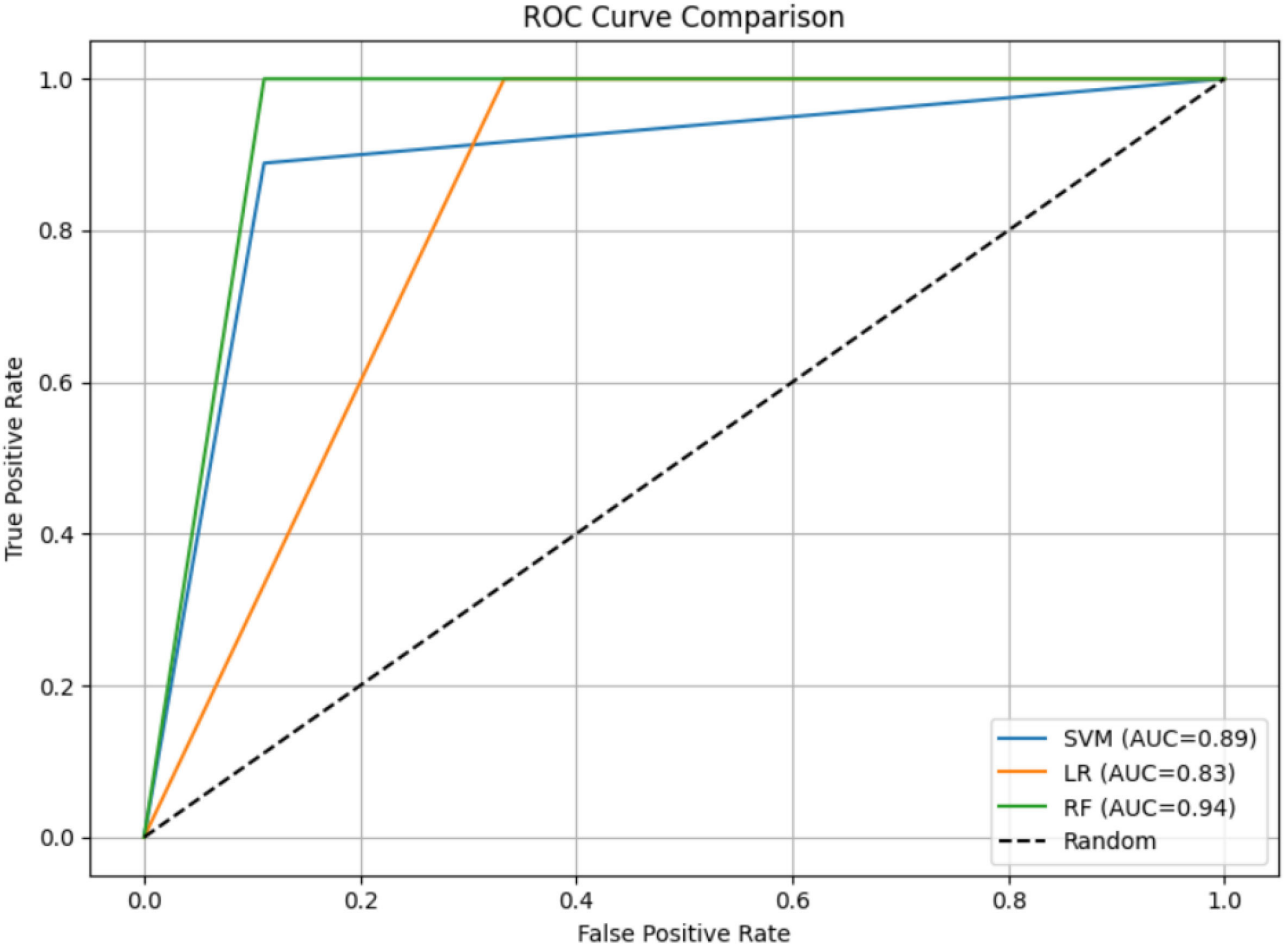
ROC curve comparing the classification performance of Random Forest (RF), Support Vector Machine (SVM), and Logistic Regression (LR) models. The RF model achieved the highest AUC of 0.94, indicating superior discriminative ability, followed by SVM with an AUC of 0.89 and LR with an AUC of 0.83. Higher AUC values represent better overall model performance.

**Figure 6:**
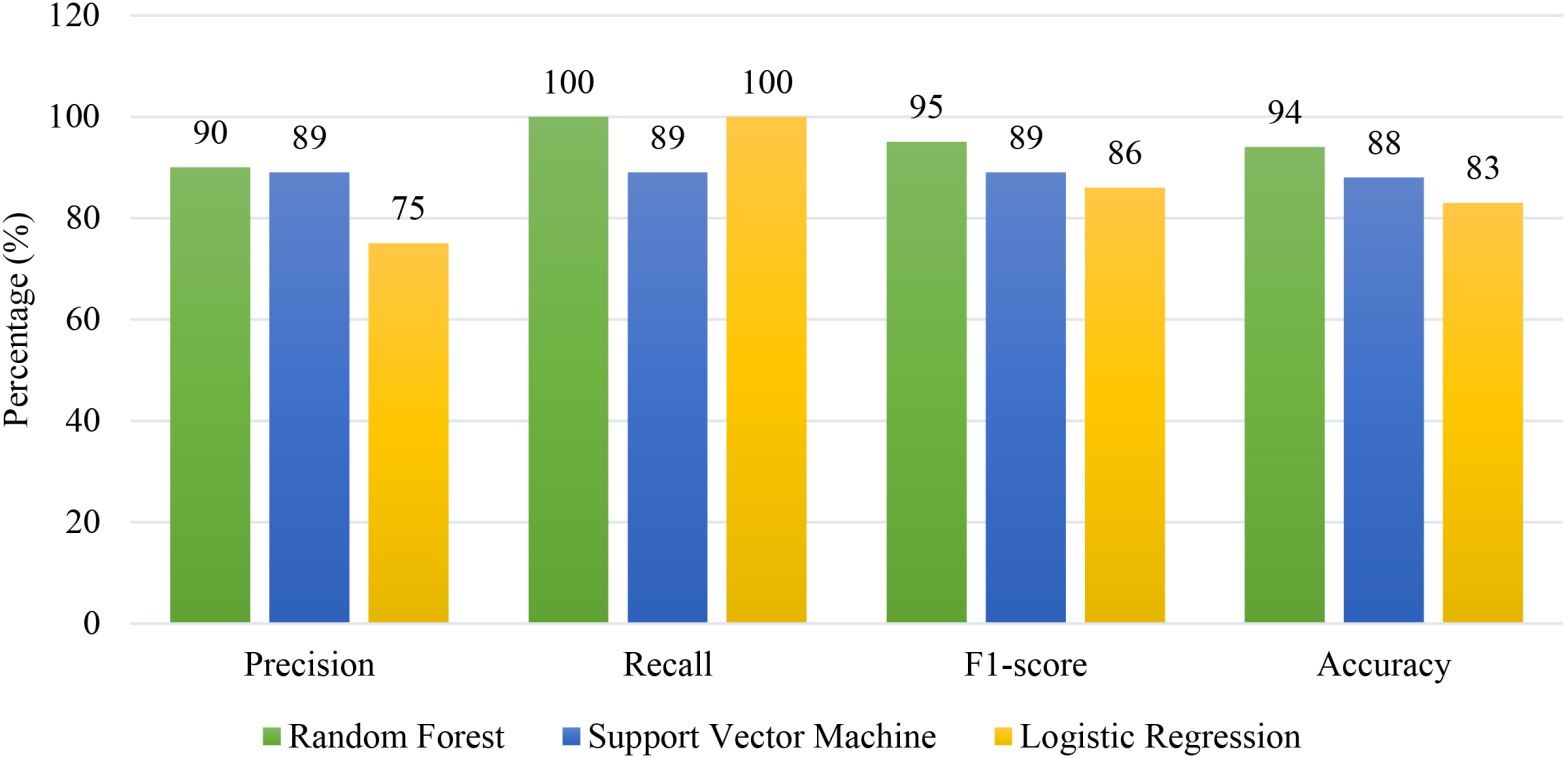
Model evaluation scores for random forest, support vector machine and logistic regression, illustrating precision, recall, F1-score and accuracy for each model.

The performance evaluation of the filtering layer and classification models was conducted on the Vanderbilt assessment tool’s symptom items. On original test data (before exclusion of overlapping), the filtering layer identified 18 ADHD-related symptom items with high accuracy of 97% perfect precision and specificity (100%), and a minimal false negative count of one. Subsequent classification of these filtered symptom items was performed using Random Forest (RF), Support Vector Machine (SVM) and Logistic Regression (LR). The RF model attained an accuracy of 94%, precision of 90%, recall of 100%, and F1-score of 0.95. The SVM model achieved an accuracy, precision, recall and F1 score of 89% respectively, while the LR model yielded slightly lower values with an accuracy of 83%, precision of 75%, and perfect recall of 100% and F1-score of 0.86. Receiver Operating Characteristics (ROC) curve analysis as shown in **Figure 5**, yielding an area under the curve (AUC) of 0.94 for RF as compared to 0.89 and 0.83 for SVM and LR, respectively. **Figure 6** summarizes the comparative evaluation metrics of these models. Per class classification matrices were given in **Table 8** with inattention encoded as class 0 and hyperactivity/impulsivity as 1.

**Table 8:** Per-class classification metrics for ADHD and Non-ADHD symptom items on the original test dataset across RF, SVM and LR classifiers.

Class	Precision	Recall	F1-score	Model
0	0.90	1	0.95	Random forest
1	1	0.89	0.94	
Accuracy			0.94	
0	0.89	0.89	0.89	SVM
1	0.89	0.89	0.89	
Accuracy			0.89	
0	0.75	1	0.86	Logistic regression
1	1	0.67	0.80	
Accuracy			0.83	

**Figure 7:**
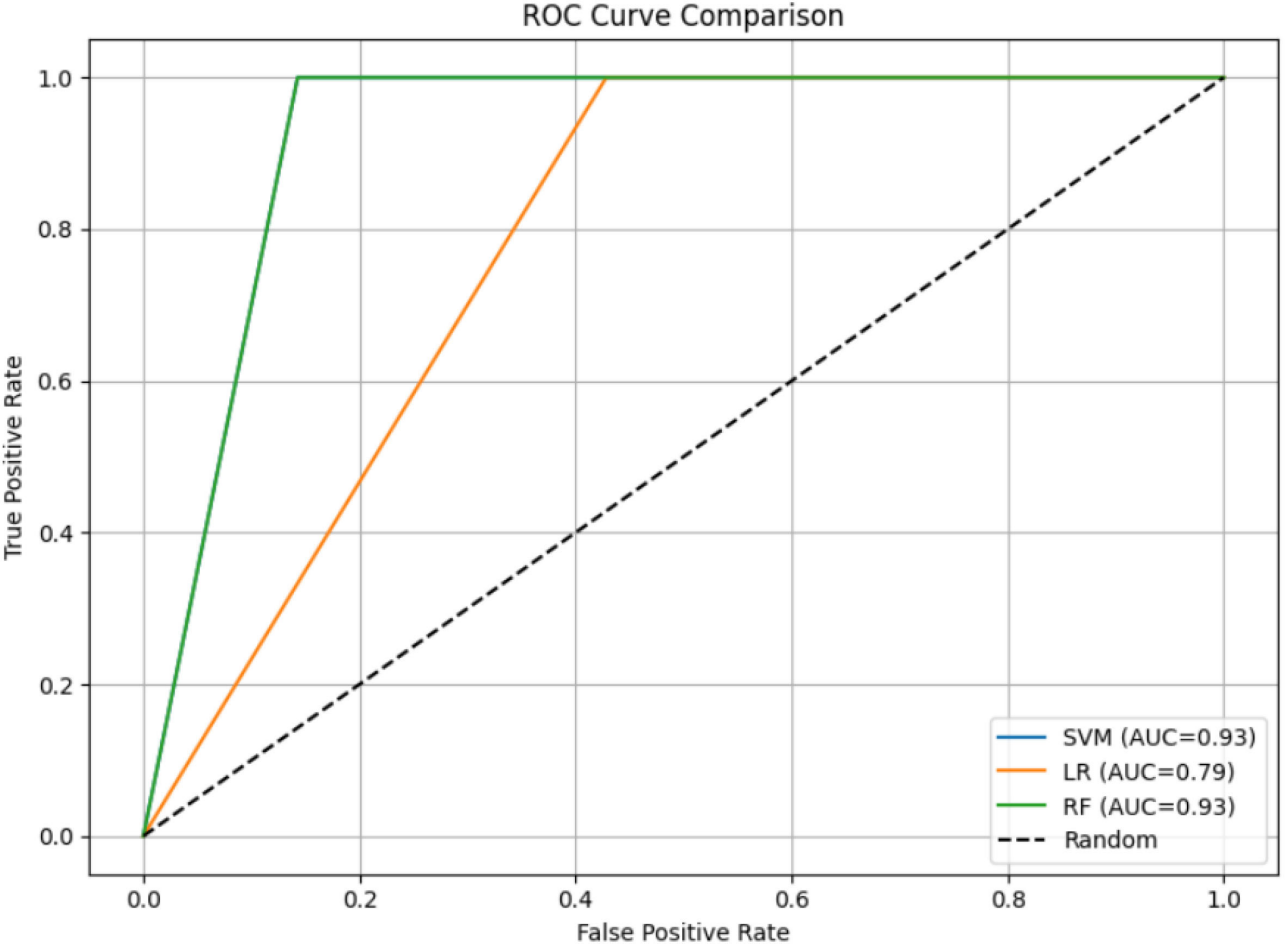
ROC curve comparing the classification performance of Random Forest (RF), Support Vector Machine (SVM), and Logistic Regression (LR) models on overlapping excluded test data. The RF and SVM model achieved the AUC of 0.93, indicating superior discriminative ability, followed by LR with an AUC of 0.79. Higher AUC values represent better overall model performance.

**Figure 8:**
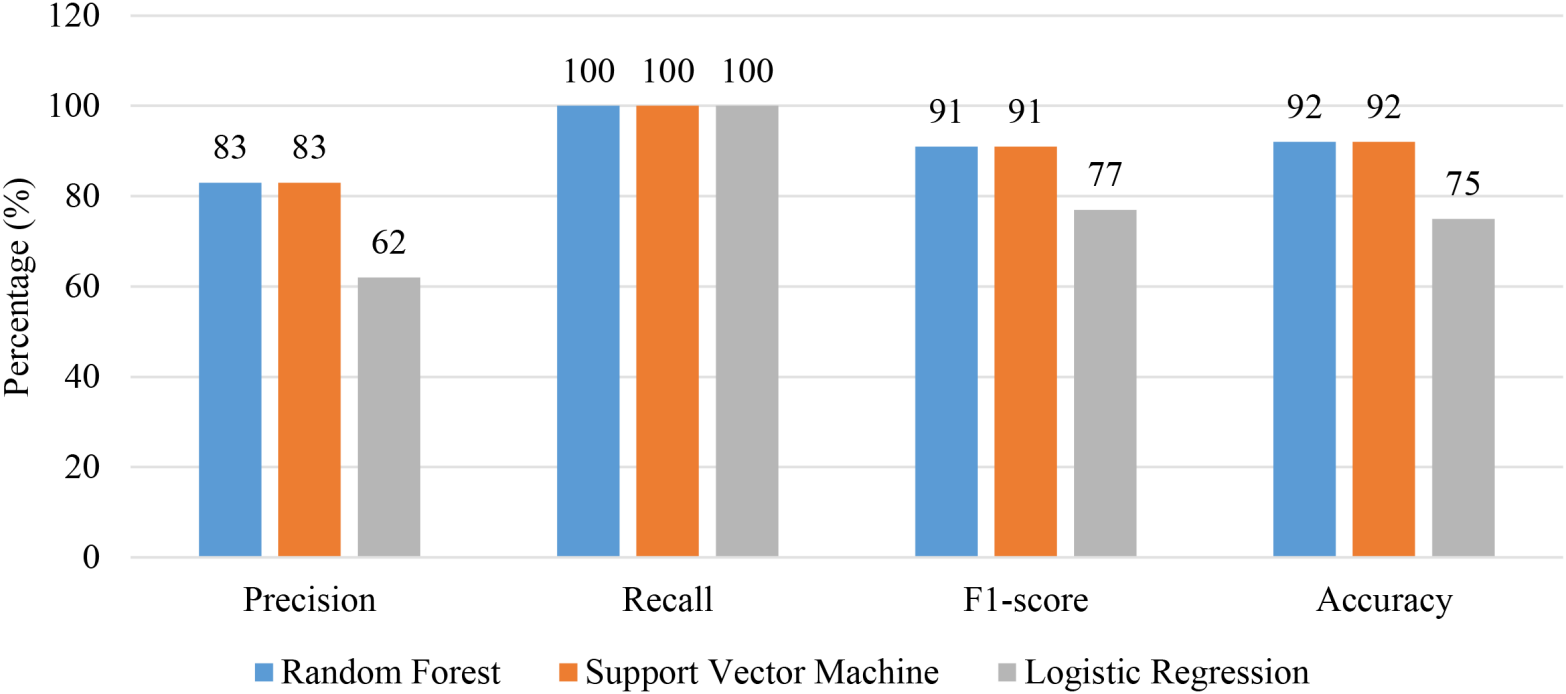
Model evaluation scores for random forest, support vector machine and logistic regression, illustrating precision, recall, F1-score and accuracy for each model using overlapping excluded test set.

On the overlapping excluded test data, the filtering layer correctly identified 12 ADHD-related symptoms with 100% precision and specificity and only one false negative. The retained items were then classified by the same three classifiers. RF and SVM yielded same performance with (accuracy of 92%, precision of 83%, recall of 100%, and F1-score of 0.91) followed by LG (accuracy of 75%, precision of 62%, recall of 100%, and F1-score of 0.77). ROC analysis shown in **Figure 7** showed the same AUC for RF and SVM (0.93) compared with LR (79). A comparative summary of model matrices has been presented in **Figure 8** with per-class classification results in **Table 9**.

**Table 9:** Per-class classification metrics for ADHD and Non-ADHD symptom items on the overlapping excluded test dataset across RF, SVM and LR classifiers.

Class	Precision	Recall	F1-score	Model
0	0.83	1	0.91	Random forest
1	1	0.86	0.92	
Accuracy			0.92	
0	0.83	1	0.91	SVM
1	1	0.86	0.92	
Accuracy			0.92	
0	0.63	1	0.78	Logistic regression
1	1	0.57	0.73	
Accuracy			0.75	

**Figure 9:**
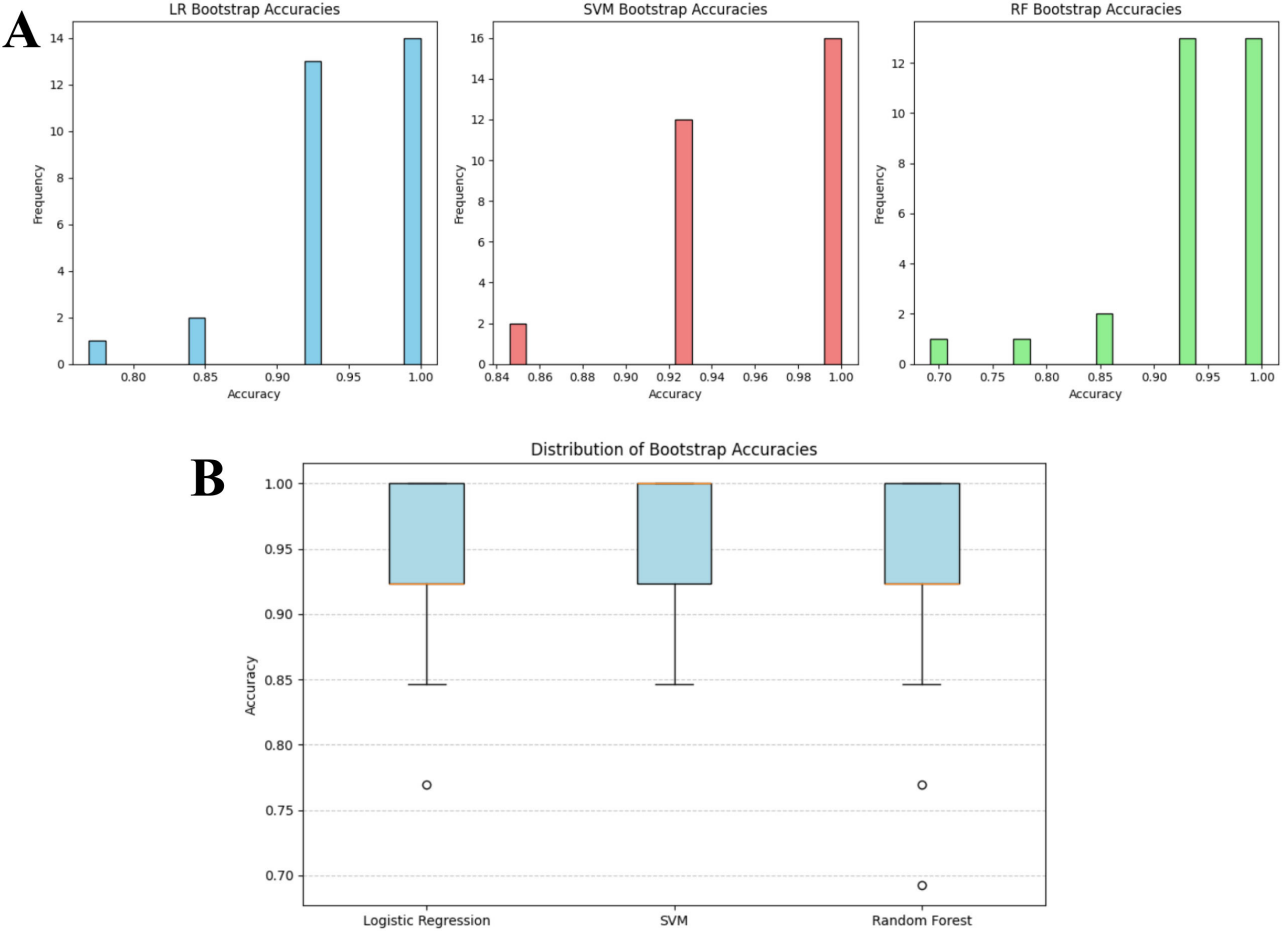
Bootstrap accuracy distributions for logistic regression, SVM and random forest on the overlap-excluded test set. **(A)** Bar plots and **(B)** box plot illustrate the stability of classifier performance across 30 bootstrap iterations. Accuracy values are concentrated in the upper performance range (LR: 0.75-1.00, SVM: 0.84-1.00, RF: 0.70-1.00), indicating limited variability and high model robustness following removal of overlapping items.

**Figure 10:**
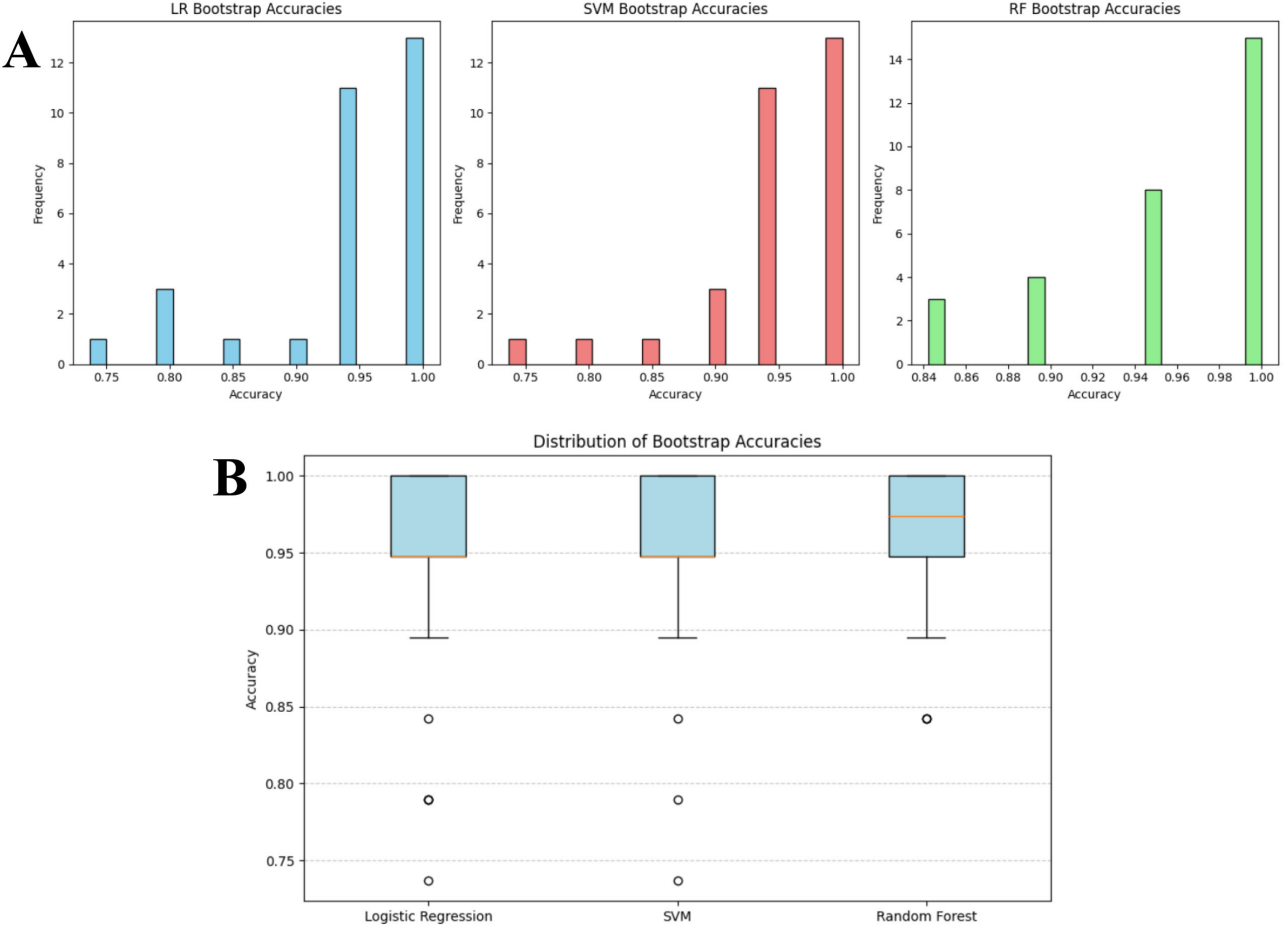
Bootstrap accuracy distributions for logistic regression, SVM and random forest on the original test set. **(A)** Bar plots and **(B)** box plot illustrate the stability of classifier performance across 30 bootstrap iterations. Accuracy values are concentrated in the upper performance range (LR and SVM: 0.75-1.00, RF: 0.84-1.00), indicating limited variability across different model performances.

**Figure 11:**
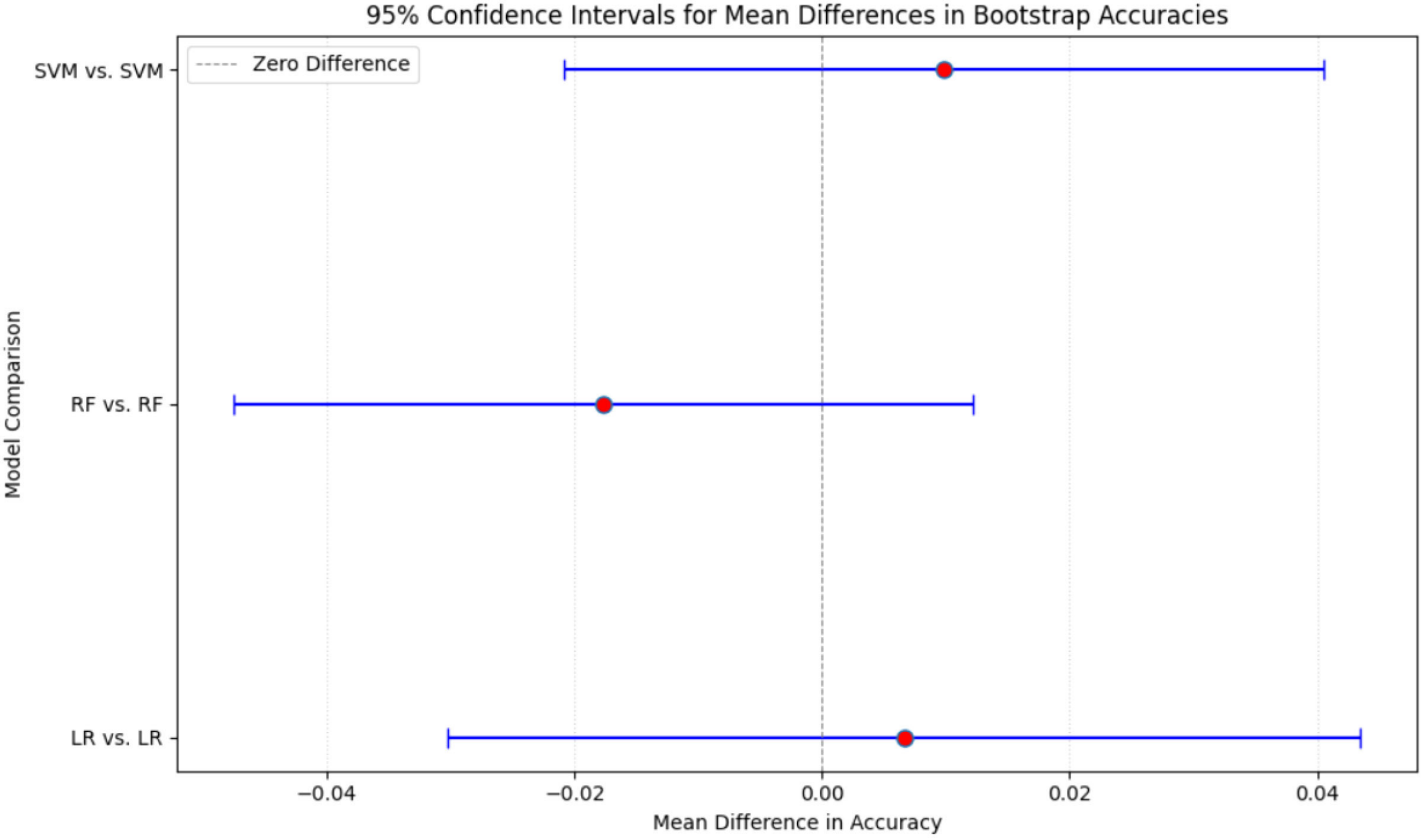
95% confidence intervals for bootstrap-based differences in model accuracy before and after overlap exclusion. The plot displays mean accuracy differences and their 95% confidence intervals for LR, RF and SVM across 30 bootstrap iterations. All confidence intervals include zero (LR: diff = 0.007, CI = [-0.030, 0.043], RF: diff = -0.018, CI = [-0.048, 0.012], SVM: diff = 0.010, CI = [-0.021, 0.041], indicating no statistically significant change in performance between the original and overlap excluded test sets. These results provide evidence that model accuracy was not inflated by lexical overlap and that the pipeline remains robust to potential data-leakage effects.

To evaluate whether the proposed pipeline was susceptible to performance inflation due to overlap between DSM-5 training statements and screening tool items, a two stage robustness analysis was conducted. First, the filtering layer and downstream classifiers were evaluated on the full test set, second potentially overlapping items were removed and the full evaluation repeated. Importantly, the filtering layer, trained to distinguish ADHD-consistent statements from Non-ADHD symptoms, demonstrated identical performance before and after overlap exclusion, indicating that its behavior was not dependent on memorized lexical content but rather on its learned decision boundary. Bootstrapping with 30 iterations was then applied separately to both datasets to quantify the stability of the downstream classifiers and t test whether overlap removal altered model performance. On the over-lap excluded test set, mean accuracies were 0.949 (SD = 0.057) for LR, 0.959 (SD = 0.048) for SVM and 0.938 (SD = 0.073) for RF. The corresponding accuracy distributions has been shown in **Figure 9A**, demonstrate that LR scores ranged primarily between 0.75 to 1.00, SVM between 0.84 to 1.00 and RF between 0.70 to 1.00, indicating moderate variability but consistently high central performance across resamples. The box plots indicated narrow interquartile ranges concentrated towards the upper end of the accuracy scale, suggesting that occasional drops in performance were rare outliers rather than systematic instability as shown in **Figure 9B**. A comparable pattern emerged when the same procedure was applied to the original test set (without removal of overlapping items). Mean accuracies were 0.942 (SD = 0.075) for LR, 0.949 (SD = 0.064) for SVM, and 0.956 (SD = 0.053) for RF, with accuracy distributions again clustered in the upper range (0.75 to 1.00 for LR and SVM, 0.84-1.00 for RF) as shown in **Figure 10A**. The consistency of boxplot medians and dispersion across both datasets indicates that model behavior was stable despite the removal of overlapping textual content (shown in **Figure 10B**). To formally assess whether model performance differed between datasets, paired t-tests on the bootstrap accuracy distributions were conducted. No statistically significant differences were observed at 5% level of significance for any classifier (LR (before and after exclusion): t = 0.367, p-value = 0.717, RF (before and after exclusion): t = -1.211, p-value = 0.236, SVM (before and after exclusion): t = 0.656, p-value = 0.517). The 95% Confidence Intervals (CIs) for the mean accuracy differences all contained zero as shown in **Figure 11**, (LR: diff = 0.007, CI = [-0.030, 0.043], RF: diff = -0.018, CI = [-0.048, 0.012], SVM: diff = 0.010, CI = [-0.021, 0.041], confirming that any fluctuations represent sampling variation rather than systematic performance shifts attributable to data leakage. These findings indicate that the classification models are robust and not driven by information leakage, and that their predictive behavior reflects genuine discriminative leaning rather than memorization of paraphrased diagnostic content.

After confirming that performance was not inflated by lexical overlap, model selection was conducted using only the overlap-excluded test set, as this dataset provides the most conservative and leakage-controlled estimate of classifier behavior. Bootstrap resampling (30 iterations) was applied to the accuracies of the three classifiers as shown in **Figure 9**, the paired t-tests were used to compare models. The results indicated no statistically significant differences in mean accuracy across models (LR vs. SVM: t = -1.278, p-value = 0.211, LR vs. RF: t = 1.000, p-value = 0.326, SVM vs. RF: t = 1.610, p-value = 0.118), a finding further supported by 95% confidence intervals that all crossed zero (LR-SVM: -0.027 to 0.006, LR-RF: -0.011 to 0.031, SVM-RF: -0.006 to 0.047). Although the difference were not statistically significant, the RF model was selected for external validation because as an ensemble method, it is well suited to capture complex, non-linear relationships among features and demonstrate greater robustness to noise and outliers through performance stabilization via aggregation across multiple decision tress (50, 60–62). Selection on the conservative (overlap-excluded) test data ensures that the chosen model reflects true generalization performance rather than artifact-driven similarity, thereby aligning with best-practice recommendations for preventing Type-I inflation in NLP based classifier evaluation.

The Vanderbilt assessment tool for ADHD strength lies in its comprehensive inclusion of all 18 DSM-5 core ADHD symptom items, which facilitates thorough symptom-level screening aligned with clinical diagnostic standards. However, alongside ADHD symptoms, Vanderbilt assessment tool incorporates approximately 28 additional items related to frequently comorbid behavioral disorders such as Oppositional Defiant Disorder (ODD) and Conduct Disorder (CD). While this broader item inclusion enhances ecological validity by accounting for complex behavioral presentations common in affected populations, it introduces challenges in specificity. The overlapping symptom domains associated with ODD and CD may complicate pure ADHD symptom discrimination. Thereby potentially inflating false positives when relaying solely on the screening tools. This represents a trade-off between comprehensive behavioral assessment and diagnostic accuracy. Careful interpretation of Vanderbilt assessment tool results in conjunction with clinical evaluation and multistage analytical frameworks is therefore essential.

### 3.3 Validation of purposed pipeline

**Figure 12:**
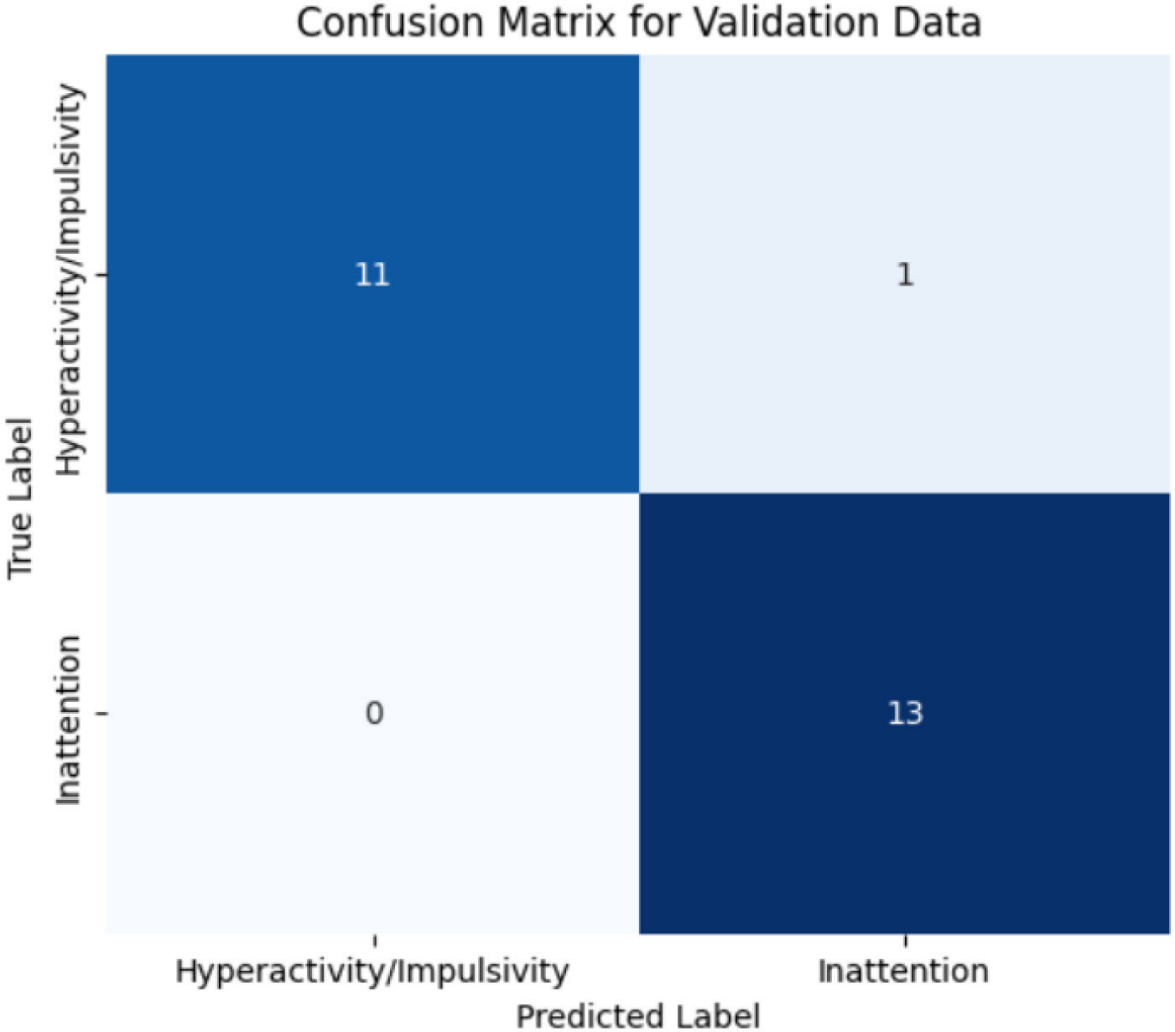
Confusion matrix for the overlap-excluded validation dataset, showing domain-level classification outcome for ADHD-related items using Random Forest classifier.

External validation was conducted using three widely-applied ADHD screening instruments: the ADHD Rating Scale, SNAP-IV and M-CHAT. Prior to model evaluation, potential textual overlap between the training and validation data has been assessed and removed to form an overlap-excluded validation subset. The filtering layer perfectly separated Non-ADHD statements, correctly rejecting all M-CHAT items and SNAP-IV item 20, 21 and 26 (Note that SNAP-IV has item 19 to 26 related to non-ADHD behaviors indicators, due to overlap exclusion only 3 items retained for evaluation) across the three screening tools with 100% specificity and precision, indicating that no non-ADHD statements progressed to the second-stage classifier. This confirms that filter layer reliably distinguished ADHD-relevant from developmentally-atypical but non-diagnostic statements prior to downstream classification. On the retained ADHD-relevant items, the selected RF classifier showed high overall accuracy, with a single pattern of domain-level misclassification. Specifically, one Hyperactivity/Impulsivity item (“*Often has difficulty playing or engaging in leisure activities quietly*”) was misclassified as belonging to the inattention domain (false positive within domain assignment). Importantly, all items were still correctly recognized as ADHD-related, the errors reflected only domain switching rather than diagnostic rejection. The corresponding confusion matrix is reported in **Figure 12**. These results show that, after removing lexically overlapping items, model performance remains stable and errors are restricted to borderline linguistic cases where behavioral phrasing may plausibly map onto either attentional or hyperactivity constructs.

The strength and limitation of these tools are closely tied to the composition of their symptom items. The ADHD Rating Scale exclusive focus on ADHD related items is a significant advantage, as it ensures high specificity for ADHD symptomatology and simplifies the filtering process, minimizing noise from unrelated behavioral domains. This focused scope likely contributes to the pipeline’s high classification accuracy and reduces complexity in symptom domain discrimination. Conversely, SNAP-IV’s inclusion of eight non-ADHD items introduces both opportunities and challenges. On one hand, this broader symptom coverage allows assessment of comorbid or overlapping behavioral issues commonly seen with ADHD, which may enhance ecological validity and clinical utility. On the other hand, the presence of non-ADHD items can reduce the specificity of screening by introducing potential for misclassification or confounding during filtering and subtype classification, as such items may amplify ambiguity between ADHD and other behavioral domains. This complexity demands more nuanced interpretation to accurately delineate symptom origins.

This analytic pipeline provides a structured way to examine the strengths and limitations of ADHD screening questionnaires in relation to DSM-5 symptom constructs. By mapping questionnaire items to their most semantically aligned DSM-5 behaviors, the approach helps assess whether a tool includes a sufficiently representative set of clinically relevant behaviors across both inattention and hyperactivity/Impulsivity domains. This is particularly important because DSM-5 specifies that diagnostic classification depends on the presence of a minimum number of symptoms within each domain (e.g., 5 out of 9). The present method does not assume that a few key items are sufficient to define ADHD, instead, it highlights where questionnaire may over or under represent certain behaviors, or where item wording may shift meaning towards adjacent constructs. In this way, the pipeline can inform refinement of existing tools, guide the development of more balanced and criterion-consistent item sets, and support researchers and clinicians in evaluating whether a screening tool adequately captures the breadth of ADHD related behaviors specified in DSM-5.

### 3.4 Limitation and future recommendations

Despite the promising outcomes, certain limitations must be acknowledged. The constrained dataset size presents a challenge, potentially restricting the model’s generalizability. Additionally, the study focused exclusively on screening tools designed for preschool-aged children. As a result, the framework’s effectiveness in evaluating tools for older age groups remain untested. To address these limitations, future efforts will focus on expanding the dataset to enhance the model’s robustness and ensure wider applicability. Moreover, integrating assessment tools tailored for different age groups will extend its relevance beyond preschool-aged-children-specific screening tools. A pivotal advancement will be transforming the model into smart, fully automated system, enabling seamless, real-time classification at scale. Furthermore, leveraging deep learning architectures will refine feature extraction, elevate classification accuracy, and strength decision-making capabilities, paving the way for a more intelligent, scalable, and universally applicable screening framework.

## 4 Conclusion

The core insights gained from the research are outlined as following:

- The investigation into conceptual overlap within ADHD symptom domains, using a multi-level similarity framework combine with an entropy-weighted method, revealed moderate overlap between symptom pairs (2 and 5) and (5 and 7) within the inattention domain, with similarity scores of 0.62 and 0.58 respectively.
- The multi-stage classification pipeline, comprising a filtering layer and machine learning classifiers (RF, SVM and LR), effectively separated ADHD-related symptom items and classified them into inattention and hyperactivity/impulsivity domains. The filtering layer demonstrated high accuracy of 97%, perfect precision and specificity in isolating DSM-5 ADHD symptoms. Among classifiers, RF achieved the best performance with 92% accuracy, 83% precision, 100% recall and an F1-score of 0.91.
- Testing the proposed pipeline suggest that, the Vanderbilt assessment tool effectively capture core ADHD symptoms while also assessing comorbidities like ODD and CD. This broad scope enhance ecological validity but many reduce diagnostic specificity for ADHD alone.
- Validation with ADHD–specific screening tools (ADHD Rating Scale and SHAP-IV) demonstrated the pipeline’s robustness. The ADHD Rating Scale ensured near-perfect classification due to its focused symptom set, while SNAP-IV’s inclusion of non-ADHD items slightly reduced subtype specificity. M-CHAT validation further confirmed the designed pipeline’s ability to exclude non-ADHD symptoms, supporting its classification precision.

## Data Availability

The original contributions presented in the study are included in the article/**Supplementary Material**. Further inquiries can be directed to the corresponding author.
