# Supplementary material for: A multi-layer similarity approach for analyzing ADHD symptomology and assessment methods considering DSM-5 diagnostic criteria
Source: Front Psychiatry. 2026 Jan 26;16:1671747. doi: 10.3389/fpsyt.2025.1671747 (PMC12884646; doi:10.3389/fpsyt.2025.1671747)
Supplement: Supplementary file 1 [file DataSheet1.docx]

**Appendix 1: Process flow of lexical similarity calculation using symptom 1 and 4 from inattention domain of ADHD**

**Similarity Matrix between:**

Symptom 1: Often fails to give close attention to details or makes careless mistakes.

Symptom 4: Often does not follow through on instructions and fails to finish tasks.

**1: After preprocessing:**

Tokens A ↓ ['often', 'fail', 'give', 'close', 'attention', 'detail', 'make', 'careless', 'mistake']

Tokens B → ['often', 'follow', 'instruction', 'fail', 'finish', 'task']

**2: Similarity Matrix after path similarity and levenshtein similarity:**

|  | **B1** | **B2** | **B3** | **B4** | **B5** | **B6** |
| --- | --- | --- | --- | --- | --- | --- |
| **A1** | 1 | 0.333 | 0.143 | 0.333 | 0.333 | 0.2 |
| **A2** | 0.333 | 0.333 | 0.143 | 1 | 0.333 | 0.2 |
| **A3** | 0.333 | 0.333 | 0.143 | 0.333 | 0.333 | 0.25 |
| **A4** | 0.333 | 0.333 | 0.143 | 0.333 | 1 | 0.2 |
| **A5** | 0.111 | 0.111 | 0.25 | 0.111 | 0.167 | 0.333 |
| **A6** | 0.167 | 0.25 | 0.167 | 0.167 | 0.167 | 0.143 |
| **A7** | 0.333 | 0.333 | 0.143 | 0.333 | 0.333 | 0.25 |
| **A8** | 0.333 | 0.333 | 0.143 | 0.333 | 0.333 | 0.2 |
| **A9** | 0.333 | 0.333 | 0.2 | 0.333 | 0.333 | 0.2 |

**3: Greedy alignment algorithm for similarity score calculation:**

|  | **B1** | **B2** | **B3** | **B4** | **B5** | **B6** |
| --- | --- | --- | --- | --- | --- | --- |
| **A1** | 1 | 0.333 | 0.143 | 0.333 | 0.333 | 0.2 |
| **A2** | 0.333 | 0.333 | 0.143 | 1 | 0.333 | 0.2 |
| **A3** | 0.333 | 0.333 | 0.143 | 0.333 | 0.333 | 0.25 |
| **A4** | 0.333 | 0.333 | 0.143 | 0.333 | 1 | 0.2 |
| **A5** | 0.111 | 0.111 | 0.25 | 0.111 | 0.167 | 0.333 |
| **A6** | 0.167 | 0.25 | 0.167 | 0.167 | 0.167 | 0.143 |
| **A7** | 0.333 | 0.333 | 0.143 | 0.333 | 0.333 | 0.25 |
| **A8** | 0.333 | 0.333 | 0.143 | 0.333 | 0.333 | 0.2 |
| **A9** | 0.333 | 0.333 | 0.2 | 0.333 | 0.333 | 0.2 |

After 1st iteration (select the highest similarity value (heighted in yellow) and drop that row and column)

|  | **B2** | **B3** | **B4** | **B5** | **B6** |
| --- | --- | --- | --- | --- | --- |
| **A2** | 0.333 | 0.143 | 1 | 0.333 | 0.2 |
| **A3** | 0.333 | 0.143 | 0.333 | 0.333 | 0.25 |
| **A4** | 0.333 | 0.143 | 0.333 | 1 | 0.2 |
| **A5** | 0.111 | 0.25 | 0.111 | 0.167 | 0.333 |
| **A6** | 0.25 | 0.167 | 0.167 | 0.167 | 0.143 |
| **A7** | 0.333 | 0.143 | 0.333 | 0.333 | 0.25 |
| **A8** | 0.333 | 0.143 | 0.333 | 0.333 | 0.2 |
| **A9** | 0.333 | 0.2 | 0.333 | 0.333 | 0.2 |

After 2nd iteration (select the highest similarity value (heighted in yellow) and drop that row and column)

|  | **B2** | **B3** | **B5** | **B6** |
| --- | --- | --- | --- | --- |
| **A3** | 0.333 | 0.143 | 0.333 | 0.25 |
| **A4** | 0.333 | 0.143 | 1 | 0.2 |
| **A5** | 0.111 | 0.25 | 0.167 | 0.333 |
| **A6** | 0.25 | 0.167 | 0.167 | 0.143 |
| **A7** | 0.333 | 0.143 | 0.333 | 0.25 |
| **A8** | 0.333 | 0.143 | 0.333 | 0.2 |
| **A9** | 0.333 | 0.2 | 0.333 | 0.2 |

After 3rd iteration (select the highest similarity value (heighted in yellow) and drop that row and column)

|  | **B2** | **B3** | **B6** |
| --- | --- | --- | --- |
| **A3** | 0.333 | 0.143 | 0.25 |
| **A5** | 0.111 | 0.25 | 0.333 |
| **A6** | 0.25 | 0.167 | 0.143 |
| **A7** | 0.333 | 0.143 | 0.25 |
| **A8** | 0.333 | 0.143 | 0.2 |
| **A9** | 0.333 | 0.2 | 0.2 |

After 4th iteration (select the highest similarity value (heighted in yellow) and drop that row and column)

|  | **B3** | **B6** |
| --- | --- | --- |
| **A5** | 0.25 | 0.333 |
| **A6** | 0.167 | 0.143 |
| **A7** | 0.143 | 0.25 |
| **A8** | 0.143 | 0.2 |
| **A9** | 0.2 | 0.2 |

After 5th iteration (select the highest similarity value (heighted in yellow) and drop that row and column)

|  | **B3** |
| --- | --- |
| **A6** | 0.167 |
| **A7** | 0.143 |
| **A8** | 0.143 |
| **A9** | 0.2 |

After 6th iteration (select the highest similarity value (heighted in yellow) and drop that row and column)

|  |
| --- |
| **A6** |
| **A7** |
| **A8** |

After 6^th^ iteration we left with no row and column. Now add up all the highest similarity scores and divide them with total number of iterations.

Similarity score = 1+1+1+0.333+0.333+0.2 / 6

**Similarity score = 0.6443**

**4: Penalty term calculation:**

The penalty was computed as the absolute difference between the token lengths of the two sentences, multiplied by the computed similarity, and divided by the maximum of the two sentence lengths. This penalty was subtracted from the word similarity score to obtain a length-normalized similarity.

Length of tokens in first sentence = 9

Length of tokens in second sentence = 6

Absolute difference between lengths = 9-6 = 3

Multiply absolute difference with similarity score = 3*0.6443 = 1.9329

Now divide the term 1.9329 by maximum of two lengths = 1.9329/9 = 0.2147

**Penalty term = 0.2147**

Subtract patently term from similarity score = 0.6443-0.2147 = 0.4296 round off (0.43)

**Adjusted similarity score = 0.43**

Adjusted similarity score is considered final lexical score.

Same process has been followed to calculate lexical similarity between each pair of symptom regarding both domains (inattention and hyperactivity/impulsivity) separately.
