# Supplementary material for: A multi-layer similarity approach for analyzing ADHD symptomology and assessment methods considering DSM-5 diagnostic criteria
Source: Front Psychiatry. 2026 Jan 26;16:1671747. doi: 10.3389/fpsyt.2025.1671747 (PMC12884646; doi:10.3389/fpsyt.2025.1671747)
Supplement: Supplementary file 2 [file DataSheet2.docx]

**Appendix 3: Process flow of syntactic similarity calculation using symptom 1 and 4 from inattention domain of ADHD**

**Similarity Matrix between:**

Symptom 1: Often fails to give close attention to details or makes careless mistakes.

Symptom 4: Often does not follow through on instructions and fails to finish tasks.

**1: After preprocessing and triplet formation:**

**Triples for sentence A ↓** [('fail', 'advmod', 'often'), ('give', 'aux', 'to'), ('fail', 'xcomp', 'give'), ('attention', 'amod', 'close'), ('give', 'dobj', 'attention'), ('to', 'pobj', 'detail'), ('give', 'conj', 'make'), ('mistake', 'amod', 'careless'), ('make', 'dobj', 'mistake')]

**Triples for sentence B →** [('have', 'advmod', 'often'), ('have', 'dobj', 'difficulty'), ('sustain', 'dobj', 'attention'), ('sustain', 'prep', 'in'), ('in', 'pobj', 'task'), ('sustain', 'conj', 'play'), ('play', 'dobj', 'activity')]

**2: Dummy Example of similarity value calculation:**

Now we take first triplet of sentence A and first triplet of sentence B:

**Heads**

**Dependents**

(‘Fail', 'advmod', 'often')

(‘Have', 'advmod', 'often')

At first step we have to compute word similarity between head of first triplet with the head of second triplet by using word similarity function. First, path-based semantic similarity was computed using Word Net’s path distance. If no valid semantic path existed between the words, or if the computed similarity was below a minimum threshold (0.1), a fallback levenshtein similarity was used. For example similarity value between heads of both triplets by using word similarity function say:

**Fail**

**Have**

**0.12**

Now we have to compute the word similarity between dependent of first triplet with the dependent of second triplet by using the same word similarity function. For example similarity value between dependents of both triplets by using word similarity function say:

**Often**

**Often**

**1**

Now take average of these 2 similarities and save in a variable name **T1** 0.12+1 / 2 = 0.56

Now we have to take similarity between triplets in cross-wise manner too for cross alignment. Compute word similarity between head of first triplet with the dependent of second triplet and wise versa by using word similarity function. For example cross-similarity value between both triplets by using word similarity function say:

**Fail**

**Often**

**0.44**

**Often**

**Have**

**0.44**

Now take average of these 2 similarities and save in a variable name **T2** 0.44+ 0.44 / 2 = 0.44

Now take average of T1 and T2 which is: 0.56+0.44 / 2 = **0.50**

Now this is the similarity score of a pair of triplet and same process followed to find the similarity matrix of all triplets.

**3: Similarity Matrix after path similarity and levenshtein similarity:**

|  | **B1** | **B2** | **B3** | **B4** | **B5** | **B6** | **B7** |
| --- | --- | --- | --- | --- | --- | --- | --- |
| **A1** | 0.500 | 0.238 | 0.222 | 0.333 | 0.267 | 0.333 | 0.250 |
| **A2** | 0.405 | 0.327 | 0.261 | 0.222 | 0.229 | 0.222 | 0.175 |
| **A3** | 0.500 | 0.405 | 0.226 | 0.333 | 0.279 | 0.333 | 0.250 |
| **A4** | 0.222 | 0.197 | 0.389 | 0.222 | 0.244 | 0.257 | 0.271 |
| **A5** | 0.389 | 0.363 | 0.392 | 0.222 | 0.257 | 0.257 | 0.271 |
| **A6** | 0.155 | 0.125 | 0.224 | 0.139 | 0.161 | 0.139 | 0.142 |
| **A7** | 0.667 | 0.571 | 0.229 | 0.333 | 0.292 | 0.375 | 0.292 |
| **A8** | 0.333 | 0.238 | 0.236 | 0.333 | 0.267 | 0.333 | 0.271 |
| **A9** | 0.500 | 0.405 | 0.240 | 0.333 | 0.279 | 0.375 | 0.312 |

**4: Greedy alignment algorithm for similarity score calculation:**

|  | **B1** | **B2** | **B3** | **B4** | **B5** | **B6** | **B7** |
| --- | --- | --- | --- | --- | --- | --- | --- |
| **A1** | 0.525 | 0.238 | 0.222 | 0.333 | 0.267 | 0.333 | 0.250 |
| **A2** | 0.405 | 0.327 | 0.261 | 0.222 | 0.229 | 0.222 | 0.175 |
| **A3** | 0.500 | 0.405 | 0.226 | 0.333 | 0.279 | 0.333 | 0.250 |
| **A4** | 0.222 | 0.197 | 0.389 | 0.222 | 0.244 | 0.257 | 0.271 |
| **A5** | 0.389 | 0.363 | 0.392 | 0.222 | 0.257 | 0.257 | 0.271 |
| **A6** | 0.155 | 0.125 | 0.224 | 0.139 | 0.161 | 0.139 | 0.142 |
| **A7** | 0.667 | 0.571 | 0.229 | 0.333 | 0.292 | 0.375 | 0.292 |
| **A8** | 0.333 | 0.238 | 0.236 | 0.333 | 0.267 | 0.333 | 0.271 |
| **A9** | 0.500 | 0.405 | 0.240 | 0.333 | 0.279 | 0.375 | 0.312 |

After 1st iteration (select the highest similarity value (heighted in yellow) and drop that row and column)

|  | **B2** | **B3** | **B4** | **B5** | **B6** | **B7** |
| --- | --- | --- | --- | --- | --- | --- |
| **A1** | 0.238 | 0.222 | 0.333 | 0.267 | 0.333 | 0.250 |
| **A2** | 0.327 | 0.261 | 0.222 | 0.229 | 0.222 | 0.175 |
| **A3** | 0.405 | 0.226 | 0.333 | 0.279 | 0.333 | 0.250 |
| **A4** | 0.197 | 0.389 | 0.222 | 0.244 | 0.257 | 0.271 |
| **A5** | 0.363 | 0.392 | 0.222 | 0.257 | 0.257 | 0.271 |
| **A6** | 0.125 | 0.224 | 0.139 | 0.161 | 0.139 | 0.142 |
| **A8** | 0.238 | 0.236 | 0.333 | 0.267 | 0.333 | 0.271 |
| **A9** | 0.405 | 0.240 | 0.333 | 0.279 | 0.375 | 0.312 |

After 2nd iteration (select the highest similarity value (heighted in yellow) and drop that row and column)

|  | **B3** | **B4** | **B5** | **B6** | **B7** |
| --- | --- | --- | --- | --- | --- |
| **A1** | 0.222 | 0.333 | 0.267 | 0.333 | 0.250 |
| **A2** | 0.261 | 0.222 | 0.229 | 0.222 | 0.175 |
| **A4** | 0.389 | 0.222 | 0.244 | 0.257 | 0.271 |
| **A5** | 0.392 | 0.222 | 0.257 | 0.257 | 0.271 |
| **A6** | 0.224 | 0.139 | 0.161 | 0.139 | 0.142 |
| **A8** | 0.236 | 0.333 | 0.267 | 0.333 | 0.271 |
| **A9** | 0.240 | 0.333 | 0.279 | 0.375 | 0.312 |

After 3rd iteration (select the highest similarity value (heighted in yellow) and drop that row and column)

|  | **B4** | **B5** | **B6** | **B7** |
| --- | --- | --- | --- | --- |
| **A1** | 0.333 | 0.267 | 0.333 | 0.250 |
| **A2** | 0.222 | 0.229 | 0.222 | 0.175 |
| **A4** | 0.222 | 0.244 | 0.257 | 0.271 |
| **A6** | 0.139 | 0.161 | 0.139 | 0.142 |
| **A8** | 0.333 | 0.267 | 0.333 | 0.271 |
| **A9** | 0.333 | 0.279 | 0.375 | 0.312 |

After 4th iteration (select the highest similarity value (heighted in yellow) and drop that row and column)

|  | **B4** | **B5** | **B7** |
| --- | --- | --- | --- |
| **A1** | 0.333 | 0.267 | 0.250 |
| **A2** | 0.222 | 0.229 | 0.175 |
| **A4** | 0.222 | 0.244 | 0.271 |
| **A6** | 0.139 | 0.161 | 0.142 |
| **A8** | 0.333 | 0.267 | 0.271 |

After 5th iteration (select the highest similarity value (heighted in yellow) and drop that row and column)

|  | **B5** | **B7** |
| --- | --- | --- |
| **A2** | 0.229 | 0.175 |
| **A4** | 0.244 | 0.271 |
| **A6** | 0.161 | 0.142 |
| **A8** | 0.267 | 0.271 |

After 6th iteration (select the highest similarity value (heighted in yellow) and drop that row and column)

|  | **B5** |
| --- | --- |
| **A2** | 0.229 |
| **A4** | 0.244 |
| **A6** | 0.161 |

After 7^th^ iteration (select the highest similarity value (heighted in yellow) and drop that row and column)

|  |
| --- |
| **A2** |
| **A6** |

After 7^th^ iteration we left with no row and column. Now add up all the highest similarity scores and divide them with total number of iterations.

Similarity score = 0.667+0.405+0.392+0.375+0.333+0.271+0.244 / 7 = 0.38

**Final Similarity score = 0.38**

Same process has been followed to calculate syntactic similarity between each pair of symptom regarding both domains (inattention and hyperactivity/impulsivity) separately.
