# Supplementary material for: A multi-layer similarity approach for analyzing ADHD symptomology and assessment methods considering DSM-5 diagnostic criteria
Source: Front Psychiatry. 2026 Jan 26;16:1671747. doi: 10.3389/fpsyt.2025.1671747 (PMC12884646; doi:10.3389/fpsyt.2025.1671747)
Supplement: Supplementary file 3 [file DataSheet3.docx]

Example sentences from inattention domain

S1: Often fails to give close attention to details or makes careless mistakes.

S2: Often has difficulty sustaining attention in tasks or play activities.

Both these sentences pass through sentence transformer model to generate 768 dimensional embedding (numerical vectors)

S1:

S2:

Compute cosine similarity between two sentences

Cosine similarity = $\frac{A.B}{|\left| A \right||\times|\left| B \right||}$

Where A.B is the dot product of vectors (sum of the products of corresponding elements). While denominator is the length of each vector. Resulting score is between -1 to +1. In case of S1 and S2 the cosine similarity is 0.49 using primary sentence transformer model.

**Figure S1:** Process flow of computing semantic similarity between sentence pairs using sentence transformer models


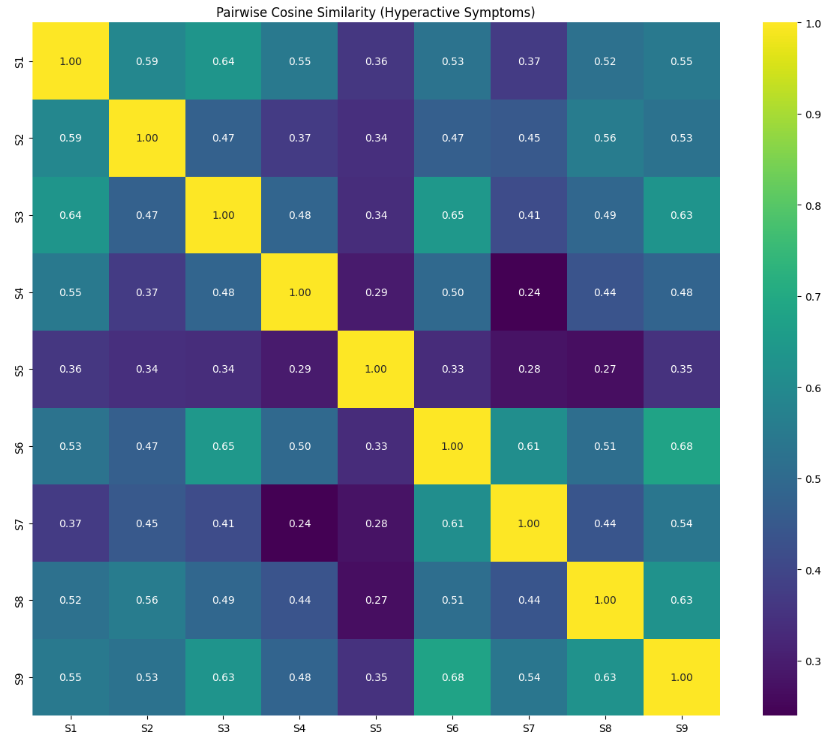


**B**


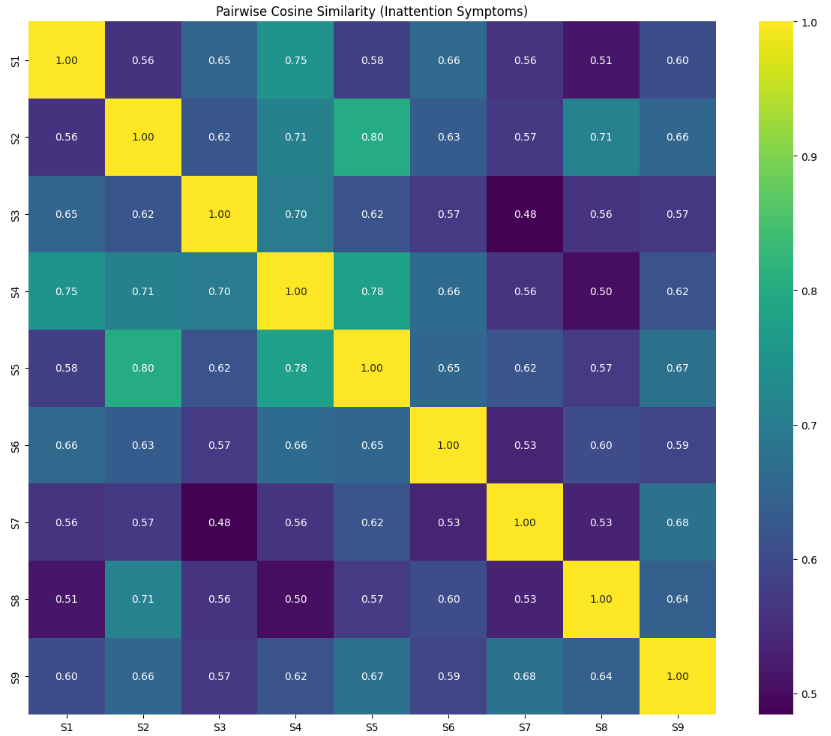


**A**

**Figure S2:** Semantic similarity heat maps for ADHD symptom domains based on sentences embedding generated by general purpose model (all-mpnet-base-v2). (A) Inattention domain: pairwise cosine similarity among symptom statements. (B) Hyperactivity/Impulsivity: pairwise cosine similarity among symptom statements. Darker shades indicate higher conceptual similarity between symptoms.


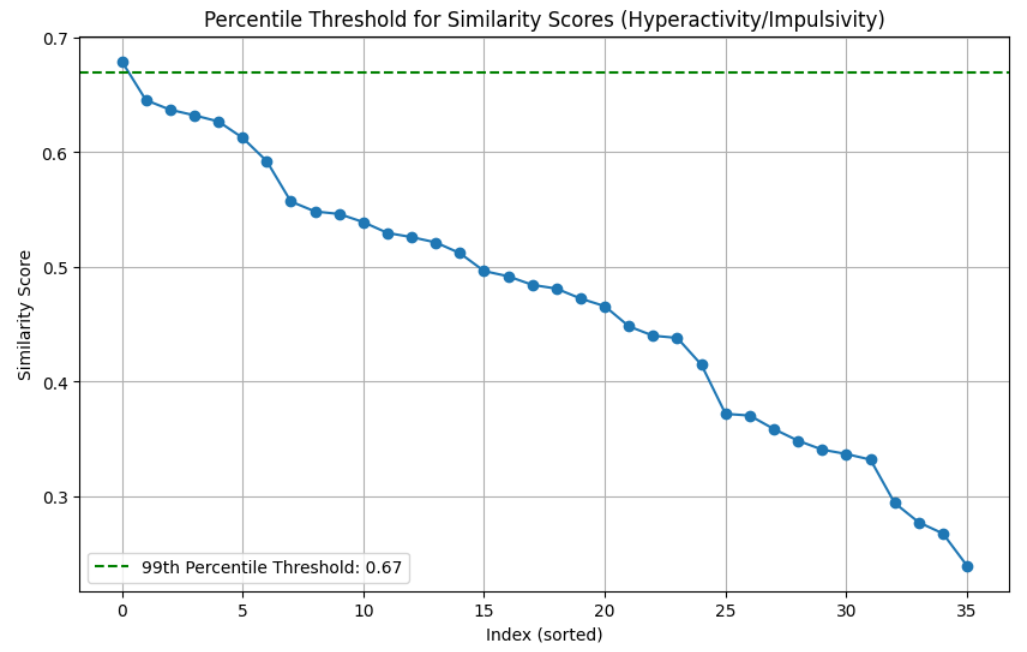


**B**


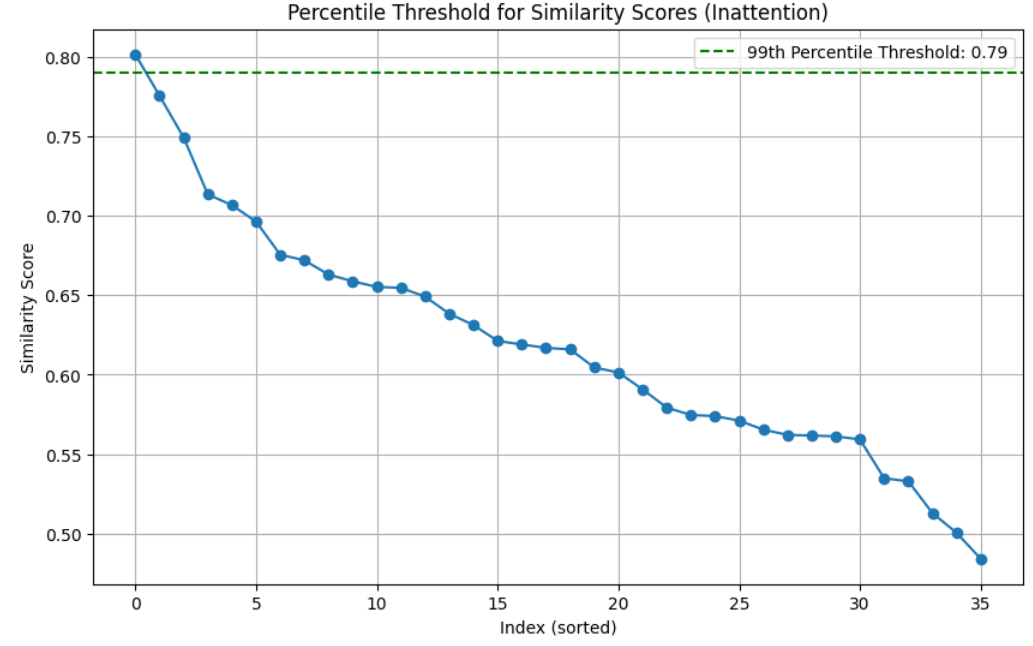


**A**

**Figure S3:** Distribution of pairwise semantic similarity scores within ADHD symptom domains using general purpose model. (A) Inattention symptoms and (B) Hyperactivity/Impulsivity symptoms. The green dashed lines indicate the top 1^st^ percentile thresholds (0.79 for inattention and 0.67 for hyperactivity/impulsivity), used to identify highly similar symptom pairs.

**Table S1: symptoms pairs exhibiting high sematic similarity based on cosine scores within each ADHD domain using general purpose model. Three pairs were identified in inattention and three in hyperactivity/impulsivity domain respectively.**

| **Sr.No** | **Pair** | **Symptom 1** | **Symptom 2** | **Similarity score** | **Domain** |
| --- | --- | --- | --- | --- | --- |
| 1 | (1,4) | Often fails to give close attention to details or makes careless mistakes | Often does not follow through on instructions and fails to finish tasks | 0.75 | Inattention |
| 2 | (2,5) | Often has difficulty in sustaining attention in tasks or play activities | Often has difficulty organizing tasks and activities | 0.80 | Inattention |
| 3 | (4,5) | Often does not follow through on instructions and fails to finish tasks | Often has difficulty organizing tasks and activities | 0.78 | Inattention |
| 4 | (1,3) | Often fidgets with or taps hands or feet, or squirms in seat. | Often runs about or climbs in inappropriate situations. | 0.64 | Hyperactivity/Impulsivity |
| 5 | (3,6) | Often runs about or climbs in inappropriate situations. | Often talks excessively. | 0.65 | Hyperactivity/Impulsivity |
| 6 | (6,9) | Often talks excessively. | Often interrupts or intrudes on others. | 0.68 | Hyperactivity/Impulsivity |


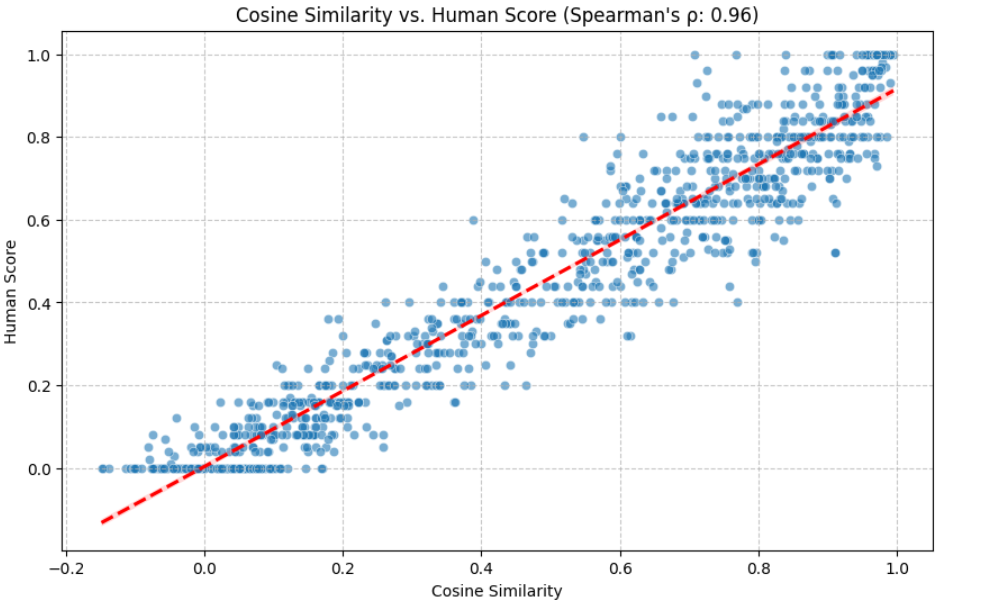


**Figure S4:** Scatter plot of primary model-generated cosine similarity scores versus human-annotated similarity scores on the STS-B dataset. Each point represents a sentence pair, with similarity values and human annotated scores, normalized between 0 and 1. The model outputs shows very strong alignment with human judgments, yielding a Spearman correlation of 0.96.
