# Supplementary material for: A multi-layer similarity approach for analyzing ADHD symptomology and assessment methods considering DSM-5 diagnostic criteria
Source: Front Psychiatry. 2026 Jan 26;16:1671747. doi: 10.3389/fpsyt.2025.1671747 (PMC12884646; doi:10.3389/fpsyt.2025.1671747)
Supplement: Supplementary file 4 [file DataSheet4.docx]

Lexical Layer

import numpy as np

from nltk.corpus import wordnet as wn

from nltk.stem import WordNetLemmatizer

from nltk.tokenize import word_tokenize

from nltk.corpus import stopwords

import Levenshtein

import nltk

import re

# Download required NLTK data

nltk.download('stopwords')

nltk.download('punkt')

nltk.download('wordnet')

nltk.download('omw-1.4')

nltk.download('punkt_tab')

nltk.download('averaged_perceptron_tagger_eng')

lemmatizer = WordNetLemmatizer()

stop_words = set(stopwords.words('english'))

# Part-of-speech tagging

def get_wordnet_pos(tag):

if tag.startswith('J'):

return wn.ADJ

elif tag.startswith('V'):

return wn.VERB

elif tag.startswith('N'):

return wn.NOUN

elif tag.startswith('R'):

return wn.ADV

else:

return wn.NOUN

# Preprocessing function

def preprocess(text):

tokens = word_tokenize(text.lower())

filtered = [word for word in tokens if word.isalpha() and word not in stop_words]

tagged = nltk.pos_tag(filtered)

lemmatized = [lemmatizer.lemmatize(word, get_wordnet_pos(pos)) for word, pos in tagged]

return lemmatized

def is_number(s):

return re.match(r'^\d+$', s) is not None

def levenshtein_sim(a, b):

return Levenshtein.ratio(a, b)

def path_similarity(word1, word2):

syns1 = wn.synsets(word1)

syns2 = wn.synsets(word2)

if not syns1 or not syns2:

return None

sim_scores = [s1.path_similarity(s2) for s1 in syns1 for s2 in syns2]

sim_scores = [s for s in sim_scores if s is not None]

return max(sim_scores) if sim_scores else None

def word_similarity(word1, word2):

path_sim = path_similarity(word1, word2)

if path_sim is None or path_sim < 0.1:

return levenshtein_sim(word1, word2)

return path_sim

def greedy_matrix_similarity(list1, list2, print_matrix=False, label1="", label2=""):

n, m = len(list1), len(list2)

sim_matrix = np.zeros((n, m))

for i in range(n):

for j in range(m):

sim_matrix[i][j] = word_similarity(list1[i], list2[j])

if print_matrix:

print(f"\nSimilarity Matrix between:\n[{label1}]\nand\n[{label2}]")

print("Tokens A →", list1)

print("Tokens B ↓", list2)

print("\nMatrix:")

print(np.round(sim_matrix, 3))

total_sim = 0

used_rows, used_cols = set(), set()

for _ in range(min(n, m)):

max_val = -1

max_i = max_j = -1

for i in range(n):

if i in used_rows: continue

for j in range(m):

if j in used_cols: continue

if sim_matrix[i][j] > max_val:

max_val = sim_matrix[i][j]

max_i, max_j = i, j

if max_val == -1:

break

total_sim += max_val

used_rows.add(max_i)

used_cols.add(max_j)

return total_sim / len(used_rows) if used_rows else 0

def penalize_for_size(psim, len1, len2):

if len1 == len2:

return 0

diff = abs(len1 - len2)

base = max(len1, len2)

return (diff * psim) / base

def lexical_similarity(sent1, sent2, verbose=False):

tokens1 = preprocess(sent1)

tokens2 = preprocess(sent2)

words1 = [t for t in tokens1 if not is_number(t)]

words2 = [t for t in tokens2 if not is_number(t)]

word_psim = greedy_matrix_similarity(

words1, words2,

print_matrix=verbose,

label1=sent1,

label2=sent2

)

word_sdpc = penalize_for_size(word_psim, len(words1), len(words2))

word_sim = word_psim - word_sdpc

final_sim = word_sim

return round(final_sim, 3)

# List of hyperactive_symptoms

hyperactive_symptoms = [

"Often fidgets with or taps hands or feet, or squirms in seat.",

"Often leaves seat in situations when remaining seated is expected.",

"Often runs about or climbs in inappropriate situations.",

"Often unable to play or engage in leisure activities quietly.",

"Is often \"on the go\", acting as if \"driven by a motor\".",

"Often talks excessively.",

"Often blurts out answers before a question has been completed.",

"Often has difficulty waiting their turn.",

"Often interrupts or intrudes on others."

]

print("\nPairwise Lexical Similarities and Token-Level Similarity Matrices:\n")

for i in range(len(hyperactive_symptoms) - 1):

for j in range(i + 1, len(hyperactive_symptoms)):

sim = lexical_similarity(

hyperactive_symptoms[i],

hyperactive_symptoms[j],

verbose=True # ← this shows the similarity matrix

)

print(f"\n Similarity between [{i+1}] and [{j+1}]: {sim}")

print("=" * 60)

Sytactic Layer

import spacy

# Load spaCy model

nlp = spacy.load("en_core_web_sm")

# Extract syntactic triples (head, dep_type, dependent) using dependency parsing

def extract_syntactic_triples(sentence):

doc = nlp(sentence)

triples = []

useful_deps = {

"nsubj", "dobj", "iobj", "pobj", "prep", "aux", "xcomp", "ccomp",

"amod", "advmod", "neg", "conj", "nmod", "attr"}

for token in doc:

# skip punctuation and self-references

if token.dep_ != "punct" and token.head != token:

dep = token.dep_.lower()

if dep in useful_deps:

head = token.head.lemma_.lower()

child = token.lemma_.lower()

triples.append((head, dep, child))

return triples

# Triple similarity

def triple_similarity(t1, t2):

v1, r1, v2 = t1

v1p, r2, v2p = t2

T1 = (word_similarity(v1, v1p) + word_similarity(v2, v2p)) / 2

T2 = (word_similarity(v1, v2p) + word_similarity(v2, v1p)) / 2

return (T1 + T2) / 2

# Main function to compute syntactic similarity between two sentences

def syntactic_similarity(sent1, sent2, verbose=False):

triples1 = extract_syntactic_triples(sent1)

triples2 = extract_syntactic_triples(sent2)

if verbose:

print(f"\nTriples for sentence 1: {triples1}")

print(f"Triples for sentence 2: {triples2}")

if not triples1 or not triples2:

return 0.0

sim_matrix = np.zeros((len(triples1), len(triples2)))

for i, t1 in enumerate(triples1):

for j, t2 in enumerate(triples2):

sim_matrix[i][j] = triple_similarity(t1, t2)

if verbose:

print("\nTriple Similarity Matrix:")

print(np.round(sim_matrix, 3))

# Greedy matching of triples

total_sim = 0

used_i, used_j = set(), set()

for _ in range(min(len(triples1), len(triples2))):

max_sim = -1

max_i = max_j = -1

for i in range(len(triples1)):

if i in used_i: continue

for j in range(len(triples2)):

if j in used_j: continue

if sim_matrix[i][j] > max_sim:

max_sim = sim_matrix[i][j]

max_i, max_j = i, j

if max_sim == -1:

break

total_sim += max_sim

used_i.add(max_i)

used_j.add(max_j)

return round(total_sim / len(used_i), 3) if used_i else 0.0

print("\n Pairwise Syntactic Similarity Matrix:\n")

for i in range(len(hyperactive_symptoms) - 1):

for j in range(i + 1, len(hyperactive_symptoms)):

sim = syntactic_similarity(hyperactive_symptoms[i], hyperactive_symptoms[j], verbose=True)

print(f"\nSyntactic Similarity between [{i+1}] and [{j+1}]: {sim}")

print("-" * 60)

semantic layer

from sentence_transformers import SentenceTransformer, util

# Load the model

model = SentenceTransformer('pritamdeka/S-Biomed-Roberta-snli-multinli-stsb')

# Encode all sentences

embeddings = model.encode(hyperactive_symptoms)

# Compute cosine similarity matrix

similarity_matrix = util.cos_sim(embeddings, embeddings).numpy()
